# Macrophages Impair TLR9 Agonist Antitumor Activity through Interacting with the Anti-PD-1 Antibody Fc Domain

**DOI:** 10.3390/cancers13164081

**Published:** 2021-08-13

**Authors:** Simone Camelliti, Valentino Le Noci, Francesca Bianchi, Chiara Storti, Francesca Arnaboldi, Alessandra Cataldo, Serena Indino, Elena Jachetti, Mariangela Figini, Mario Paolo Colombo, Andrea Balsari, Nicoletta Gagliano, Elda Tagliabue, Lucia Sfondrini, Michele Sommariva

**Affiliations:** 1 Dipartimento di Scienze Biomediche per la Salute, Università degli Studi di Milano, Via Mangiagalli 31, 20133 Milan, Italy; simone.camelliti@unimi.it (S.C.); valentino.lenoci@unimi.it (V.L.N.); francesca.bianchi1@unimi.it (F.B.); chiara.storti@unimi.it (C.S.); francesca.arnaboldi1@unimi.it (F.A.); serena.indino@unimi.it (S.I.); andrea.balsari@unimi.it (A.B.); nicoletta.gagliano@unimi.it (N.G.); lucia.sfodnrini@unimi.it (L.S.); 2 U.O. Laboratorio di Morfologia Umana Applicata, IRCCS Policlinico San Donato, Piazza Edmondo Malan 2, 20097 Milan, Italy; 3 Molecular Targeting Unit, Department of Research, Fondazione IRCCS Istituto Nazionale dei Tumori, Via Amadeo 42, 20133 Milan, Italy; alessandra.cataldo@istitutotumori.mi.it (A.C.); elda.tagliabue@istitutotumori.mi.it (E.T.); 4 Molecular Immunology Unit, Department of Research, Fondazione IRCCS Istituto Nazionale dei Tumori, Via Amadeo 42, 20133 Milan, Italy; elena.jachetti@istitutotumori.mi.it (E.J.); mario.colombo@istitutotumori.mi.it (M.P.C.); 5 Dipartimento di Ricerca Applicata e Sviluppo Tecnologico, Fondazione IRCCS Istituto Nazionale dei Tumori, Via Amadeo 42, 20133 Milan, Italy; mariangela.figini@istitutotumori.mi.it

**Keywords:** ovarian cancer, Toll-like receptor 9 (TLR9), CpG oligodeoxynucleotides (CpG-ODNs), programmed cell death 1 (PD-1), macrophages, Fc receptors

## Abstract

**Simple Summary:**

We evaluated the contribution of macrophages to the effect of combinatorial immunotherapeutic treatments based on TLR9 stimulation (with CpG-ODNs) and PD-1 blockade in an ovarian cancer preclinical model. We observed a strong reduction in the antitumor efficacy of a TLR9 agonist upon anti-PD-1 antibody administration. Specifically, we found that TLR9-stimulated macrophages, through interacting with the fragment crystallizable (Fc) domain of the anti-PD-1 antibody, acquire an immunoregulatory phenotype leading to dampening of CpG-ODN antitumor effect. Since the stimulation of macrophage TLRs can be achieved not only by synthetic agonists but also by molecules present in the tumor microenvironment, the data we are presenting may represent another possible mechanism of anti-PD-1 antibody therapy resistance. Indeed, it is possible that when delivered as a monotherapy, anti-PD-1 antibody Fc domain may interact with macrophages in which TLR signaling has already been triggered by endogenous ligands, mirroring the biological effects described in the present study.

**Abstract:**

Background. A combination of TLR9 agonists and an anti-PD-1 antibody has been reported to be effective in immunocompetent mice but the role of innate immunity has not yet been completely elucidated. Therefore, we investigated the contribution of the innate immune system to this combinatorial immunotherapeutic regimens using an immunodeficient mouse model in which the effector functions of innate immunity can clearly emerge without any interference from T lymphocytes. Methods. Athymic mice xenografted with IGROV-1 human ovarian cells, reported to be sensitive to TLR9 agonist therapy, were treated with cytosine–guanine (CpG)-oligodeoxynucleotides (ODNs), an anti-PD-1 antibody or their combination. Results. We found that PD-1 blockade dampened CpG-ODN antitumor activity. In vitro studies indicated that the interaction between the anti-PD-1 antibody fragment crystallizable (Fc) domain and macrophage Fc receptors caused these immune cells to acquire an immunoregulatory phenotype, contributing to a decrease in the efficacy of CpG-ODNs. Accordingly, in vivo macrophage depletion abrogated the detrimental effect exerted by the anti-PD-1 antibody. Conclusion. Our data suggest that if TLR signaling is active in macrophages, coadministration of an anti-PD-1 antibody can reprogram these immune cells towards a polarization state able to negatively affect the immune response and eventually promote tumor growth.

## 1. Introduction

Toll-like receptors (TLRs) are receptors that are expressed by immune and nonimmune cells and are able to recognize molecules released by pathogens, i.e., so-called pathogen-associated molecular patterns (PAMPs), or produced by normal or malignant stressed or dead cells, defined as danger-associated molecular patterns (DAMPs). The engagement of TLRs by their cognate ligands provokes a potent innate immune response that, in turn, leads to the activation of adaptive immunity [1]. Due to their ability to stimulate the immune system, TLR agonists have been extensively investigated for their capability to treat different types of cancer [2]. In particular, many preclinical and clinical studies have evaluated the therapeutic potential of synthetic agonists of TLR9, an intracellular TLR for unmethylated cytosine–guanine (CpG) DNA motifs present in bacterial genomes [3]. However, the initial enthusiasm was dampened by the results of several clinical trials showing that TLR9 agonists exert a modest antitumor effect [3,4]. There is a plethora of possible mechanistic explanations for this lack of efficacy, most of which have not been completely elucidated yet. For example, the route and frequency of TLR9 agonist administration appear to be crucial factors in the ability of this immunotherapy to lead to a positive outcome. Since TLR stimulation preferentially affects innate immune cells that need to be locally and continuously stimulated to exert their optimal therapeutic effects, locoregionally and repeated administration of a TLR9 ligand was reported to have a better effect than systemic delivery of the ligand [5,6]. It has been observed that intratumor and a daily injection of CpG-oligodeoxynucleotides (ODNs) can control the growth of IGROV-1 human ovarian carcinoma cells orthotopically xenografted into athymic nude mice, indicating the importance of innate effector cell activation at the tumor bed [6,7,8]. However, these studies also showed that modifications of the treatment schedule were not sufficient to determine a complete and lasting curative effect, suggesting the co-occurrence of different immunological processes that restrain CpG-ODN activity, for example, autotuning of the immune system.

After an initial phase of stimulation, immune cells trigger negative feedback mechanisms that, although it may appear counterintuitive, are necessary to maintain an appropriate balance during the immune cell response to avoid immune cell overactivation and activation-induced cell death (AICD) and to protect the host against exaggerated inflammatory processes [9]. Indeed, different inhibitory regulatory mechanisms established after TLR activation have been identified and characterized [10]. For instance, it has been reported that TLR9 stimulation can induce the expression of the immune checkpoint receptor programmed death-1 (PD-1) on different immune cells, such as natural killer (NK) cells [11], monocytes [12] and B lymphocytes [13].

PD-1 is well known to inhibit T lymphocyte effector functions upon interaction with programmed death-ligand 1 and 2 (PD-L1 and PD-L2) [14]. Physiologically, the PD-1/PD-Ls pathway is fundamental for maintaining self-tolerance and preventing autoimmunity [15], and tumor cells exploit this pathway to escape immune elimination [16]. In the last decade, antibodies targeting PD-1 were proven to exhibit antitumor activity in a broad spectrum of cancers [16,17]. These results likely reflect a reversal of the exhaustion state of tumor-specific T cells and the ensuing enhancement of antitumor adaptive immunity [16,17,18]. In addition to T lymphocytes, innate immune cells can express PD-1, whose expression is associated with the development of cellular exhaustion [19]. Blocking PD-1 on innate immune cells may also restore their effector functions. Indeed, there is some evidence that PD-1 blockade can also be efficacious in tumor-bearing immunodeficient mice [11,20,21], suggesting that the innate immune system actively contributes to the ultimately antitumor effect of anti-PD-1 antibodies.

Therefore, TLR agonists and anti-PD-1 antibodies may be extremely effective when combined. Blockade of PD-1 signaling may enhance the immunostimulatory effects of TLR agonists, and TLR ligation may improve the effect of PD-1/PD-L1 blockade by restoring the effector functions of tumor-infiltrating immune cells. In this regard, it has been demonstrated that intratumorally (i.t.) delivered TLR9 agonists synergize with an anti-PD-1 antibody in murine head and neck [22] and bladder carcinoma models [23] and that i.t. injected CpG-ODNs abrogate resistance to anti-PD-1 therapy in a preclinical CT26 colon cancer model, leading to tumor rejection [24].

Since innate immune cells are the main targets of TLR agonists, they are likely to participate to the ultimate effects of this type of combinatorial treatment, but in the aforementioned studies performed in immunocompetent mice, the role played by the innate immune system could have been obscured by the presence of T lymphocytes. Therefore, to investigate the contribution of the innate immune system to the effect of combinatorial immunotherapeutic treatments based on TLR9 stimulation and PD-1 blockade, we utilized athymic nude mice, in which the effector functions of innate immune cells can clearly emerge without any interference from T lymphocytes, xenografted with IGROV-1 human ovarian cancer cells. This tumor histotype is generally considered poorly responsive to anti-PD-1 antibody therapy [25] and may benefit from novel combinatorial therapeutic strategies. Moreover, it is suitable for locoregional therapy, which, as mentioned above, is the optimal route of administration for TLR ligands.

Unexpectedly, we observed that blocking PD-1 on innate immune cells impaired the therapeutic activity of CpG-ODNs. Subsequent analyses suggested that macrophages that are stimulated by TLR9 agonists and interact with the fragment crystallizable (Fc) domain of the anti-PD-1 antibody can acquire an immunoregulatory phenotype leading to dampening of the antitumor effect of CpG-ODNs.

## 2. Materials and Methods

### 2.1. Reagents and Antibodies

The purified phosphorothioated TLR-9 agonist ODN1826 (5′-TCCATGACGTTCCTGACGTT-3′), which contains CpG motifs (CpG-ODNs), was synthesized by TriLink Biotechnologies (San Diego, CA, USA). Susceptibility to DNase digestion was diminished by phosphorothioate modification, prolonging the in vivo half-life of the agonist. Rat anti-mouse PD-1 (clone RMP1-14), hamster anti-mouse PD-1 (clone J43), rat IgG2a isotype control (clone 2A3) and rat anti-mouse CD16/32 (clone 2.4G2) antibodies were purchased from BioXCell (West Lebanon, NH, USA). A rat anti-mouse PD-1 (clone RMP1-14) Fc Silent™ antibody was purchased from Absolute Antibody (Redcar, Cleveland, UK). Liposomal clodronate was purchased from ClodronateLiposomes.com (Amsterdam, The Netherlands). An anti-mouse PD-1 F(ab)_2_ (clone RMP1-14, BioXCell, West Lebanon, NH, USA) antibody was generated as previously described [26]. ^51^Cr (1 mCi) was purchased from PerkinElmer (Waltham, MA, USA).

### 2.2. Cell Culture

The IGROV-1 human ovarian cancer cell line (kind gift from Dr. J. Benard, Institute Gustave Roussy, Villejuif, France), 4T1 mouse mammary tumor cell line (American Type Culture Collection, ATCC, Manassas, VA, USA), EL-4 and YAC-1 mouse lymphoma tumor cell lines (ATCC, Manassas, VA, USA) were cultured in RPMI 1640 medium (Thermo Fisher Scientific Inc., Waltham, MA, USA) supplemented with 10% fetal bovine serum (FBS, Thermo Fisher Scientific Inc., Waltham, MA, USA) and 2 mM glutamine (Thermo Fisher Scientific Inc., Waltham, MA, USA). The B16 mouse melanoma cell line (ATCC, Manassas, VA, USA) and RAW264.7 macrophage-like cell line (ATCC, Manassas, VA, USA) were maintained in DMEM (Thermo Fisher Scientific Inc., Waltham, MA, USA) supplemented with 10% FBS (Thermo Fisher Scientific Inc., Waltham, MA, USA) and 2 mM glutamine (Thermo Fisher Scientific Inc., Waltham, MA, USA). All the cell lines were routinely maintained at 37 °C in a 5% CO_2_ atmosphere. The cell lines were authenticated by the Genomic Facility at Fondazione IRCCS Istituto Nazionale dei Tumori (Milan, Italy) and regularly tested for Mycoplasma using a specific kit (MycoAlert^TM^ Mycoplasma Detection Kit, Lonza Group Ltd., Basel, Switzerland).

### 2.3. Mice and In Vivo Studies

Eight-week-old female athymic nude mice (Charles River Laboratories, Calco, Italy) were intraperitoneally (i.p.) injected with 2.5 × 10^6^ IGROV-1 cells in 0.2 mL of saline. The mice were injected i.p. with CpG-ODNs at a dose of 20 µg/mouse for 5 days/week for 3 weeks starting 5 or 7 days after tumor cell injection, injected i.p. with a monoclonal anti-mouse PD-1 antibody (clone RMP1-14 or J43, BioXCell, West Lebanon, NH, USA) at a dose of 200 µg/mouse twice a week for 3 weeks starting 7, 8 or 13 days after tumor cell injection, or administered both treatments in combination. Control mice received saline. To deplete macrophages, IGROV-1 cell-xenografted mice were treated i.p. with 400 μL of liposomal clodronate three days after tumor cell injection. The mice were then treated with CpG-ODNs and an anti-PD-1 antibody as described above. The mice in the experimental groups were inspected daily and weighed three times/week, and animals were individually sacrificed by cervical dislocation at the first evidence of ascites. The percentage of disease-free mice in each treatment group was estimated by the Kaplan–Meier product-limit method and compared with the log-rank (Mantel–Cox) test.

For in vivo studies in immunocompetent models, C57BL/6 and Balb/c mice (Charles River Laboratories, Calco, Italy) were subcutaneously (s.c.) injected with 1 × 10^6^ B16 melanoma cells or 4T1 breast cancer cells. The mice were peritumorally (p.t.) injected with 200 µg/mouse anti-mouse PD-1 antibody (clone RMP1-14) twice a week and 20 µg/mouse CpG-ODNs 5 days/week alone or in combination after tumors became palpable. The control group received saline. Tumor mass was measured with a caliper, and tumor volume (mm^3^) was calculated as [long diameter × (short diameter)^2^/2].

The mice were housed in the Animal Facility of the Fondazione IRCCS Istituto Nazionale dei Tumori (Milan, Italy) in a thermostatically maintained animal house on a 12-h light/dark cycle and given ad libitum access to food and water. The animal experiments were authorized by the Institutional Animal Welfare Body and the Italian Ministry of Health and performed in accordance with national law (D.lgs 26/2014) and the Guidelines for the Welfare of Animals in Experimental Neoplasia [27]. At the end of each experiment, tumors were harvested for subsequent analyses.

### 2.4. Flow Cytometry Analysis

Eight-week-old female athymic nude mice (Charles River Laboratories, Calco, Italy) were treated with saline or CpG-ODNs delivered i.p. at a dose of 20 µg/mouse for 4 consecutive days. At the end of treatment, the mice were individually sacrificed by cervical dislocation. After sacrifice, peritoneal cells were obtained by peritoneal lavage as previously described with slight modifications [28]. The suspended cells were directly stained for 30 min at 4 °C in PBS containing 1% Bovine Serum Albumin (BSA, Merk, Darmstadt, Germany) with anti-mouse CD45-FITC (eBioscience-Thermo Fisher Scientific Inc., Waltham, MA, USA) and anti-mouse PD-1-APC (eBioscience-Thermo Fisher Scientific Inc., Waltham, MA, USA) antibodies. To prevent nonspecific binding to mouse Fc receptors, the cells were also incubated with a purified rat anti-mouse CD16/CD32 monoclonal antibody (eBioscience-Thermo Fisher Scientific Inc., Waltham, MA, USA). Analyses were performed by gating live CD45^+^ cells after doublet exclusion.

To evaluate PD-1 expression in RAW264.7 macrophages, cells were seeded in 12-well plates (Corning, Glendale, AZ, USA) at a density of 8 × 10^5^ cells/well. The cells were then stimulated with 1 µM CpG-ODNs or left untreated for 6 h. At the end of treatment, the RAW264.7 cells were detached from plates using TrypLE™ Express Enzyme (Thermo Fisher Scientific Inc., Waltham, MA, USA) and incubated with 10 μg/mL anti-mouse PD-1 antibody (clone RMP1-14, BioXCell, West Lebanon, NH, USA) for 30 min at 4 °C. After washing, the cells were incubated with FITC-conjugated anti-rat IgG (H+L) antibody, mouse serum adsorbed (KPL, SeraCare Life Sciences, Milford, MA, USA) for 30 min at 4 °C. Samples were then acquired using a FACSCanto flow cytometer (BD Biosciences, San Jose, CA, USA) and analyzed with FlowJo software (FlowJo LCC, Ashland, OR, USA).

To evaluate CD80, CD86 and CD206 expression in RAW264.7 cell line, cells were culture and treated as described above. Cells were then incubated with anti-mouse CD80-PE (BD Pharmingen, San Diego, CA, USA, clone 16-10A1), anti-mouse CD86-APC-R700 (BD Biosciences, San Jose, CA, USA, clone GL1) or with anti-mouse CD206-PE (Elabscience, Houston, TX, USA, clone C068C2) for 30 min at 4 °C. To prevent nonspecific binding to mouse Fc receptors, the cells were also incubated with a purified rat anti-mouse CD16/CD32 monoclonal antibody (eBioscience-Thermo Fisher Scientific Inc., Waltham, MA, USA). After washing, samples were then acquired using a BD FACSCelesta™ Cell Analyzer (BD Biosciences, San Jose, CA, USA) and analyzed with FlowJo software (FlowJo LCC, Ashland, OR, USA). Analyses were performed by gating live cells after doublet exclusion.

The ability of anti-PD-1 antibody F(ab)_2_ to bind PD-1 was evaluated by flow cytometry. EL-4 mouse lymphoma cells, reported to express high level of PD-1 [29], were stained with 10 μg/mL anti-mouse PD-1 antibody (clone RMP1-14) (BioXCell, West Lebanon, NH, USA) or anti-mouse PD-1 antibody F(ab)_2_ for 30 min at 4 °C. To prevent nonspecific binding to mouse Fc receptors, the cells were also incubated with a purified rat anti-mouse CD16/CD32 monoclonal antibody (eBioscience-Thermo Fisher Scientific Inc., Waltham, MA, USA). After washing, the cells were incubated with FITC-conjugated anti-rat IgG (H+L) antibody, mouse serum adsorbed (KPL, SeraCare Life Sciences, Milford, MA, USA) for 30 min at 4 °C. Samples were then acquired using a BD FACSCelesta™ Cell Analyzer (BD Biosciences, San Jose, CA, USA) and analyzed with FlowJo software (FlowJo LCC, Ashland, OR, USA). Analyses were performed by gating live cells after doublet exclusion.

### 2.5. Immunohistochemistry and Immunofluorescence

Eight-week-old female athymic nude mice (Charles River Laboratories, Calco, Italy) were i.p. injected with 2.5 × 10^6^ IGROV-1 cells in 0.2 mL of saline. The mice were treated with a monoclonal anti-mouse PD-1 antibody (clone RMP1-14) delivered i.p. at a dose of 200 µg/mouse 5 times at two- or three-day intervals. Treatment started the day after tumor cell injection. Control mice received saline. In another experimental setting, eight-week-old female athymic nude mice (Charles River Laboratories, Calco, Italy) were i.p. injected with 2.5 × 10^6^ IGROV-1 cells in 0.2 mL of saline. Mice were treated i.p. with CpG-ODNs at a dose of 20 µg/mouse for 5 days/week for 2 weeks or with CpG-ODN in combination with a monoclonal anti-mouse PD-1 antibody (clone RMP1-14) at a dose of 200 µg/mouse twice a week for 2 weeks. Treatments started 7 days after tumor cell injection. The animals were individually sacrificed by cervical dislocation at the first evidence of ascites, and tumors were collected for immunohistochemical or immunofluorescence analysis.

Tumor samples were fixed in 10% buffered formalin, embedded in paraffin and sectioned (4 µm thick). The sections were deparaffinized and rehydrated. For immunohistochemical analysis, antigen unmasking was performed using Tris/EDTA buffer (pH 9) (Novocastra, Leica Microsystems, Buffalo Grove, IL, USA) in a PT Link Dako (Dako, Agilent, Santa Clara, CA, USA) unit at 98 °C for 30 min. The sections were then brought to room temperature (RT) and washed in PBS. After neutralization of endogenous peroxidase activity with 3% H_2_O_2_ and blocking of Fc with a specific protein (Novocastra, Leica Microsystems, Buffalo Grove, IL, USA), the samples were incubated with an anti-mouse arginase I (ArgI) primary antibody (Genetex International Corp., Irvine, CA, USA) for 1 h at RT. Staining was visualized with a polymer detection kit (Novocastra, Leica Microsystems, Buffalo Grove, IL, USA) and 3-amino-9-ethylcarbazole (AEC) substrate-chromogen (Dako, Agilent, Santa Clara, CA, USA). The slides were counterstained with Harris hematoxylin (Diapath, Martinengo, Italy). The sections were analyzed under a Leica DM2000 optical microscope (Leica Microsystems, Buffalo Grove, IL, USA), and microphotographs were collected using a Leica DFC320 digital camera (Leica Microsystems, Buffalo Grove, IL, USA), as previously described [26,30,31]. For immunofluorescence analysis, antigen retrieval was performed in 0.01 M citrate buffer (pH 6) in autoclave at 90 °C for 20 min. Autofluorescence was quenched by incubating the tumor section with 1M NaBH_4_ solution in PBS for 10 min at RT. Unspecific binding site saturation, performed at RT for 1 h with PBS containing 3% BSA, was followed by incubation with anti-mouse CD206 (BioLegend, San Diego, CA, USA) or anti-mouse IL-10-FITC (BD Biosciences, San Jose, CA, USA), overnight at 4 °C. For CD206 staining, goat anti-rat AlexaFluor 488 (Thermo Fisher Scientific Inc., Waltham, MA, USA) was utilized as a secondary antibody. Nuclei were counterstained with 4′,6-Diamidino-2-Phenylindole Dihydrochloride (DAPI, Thermo Fisher Scientific Inc., Waltham, MA, USA) for 10 min at RT. Coverslips were mounted on glass slides using ProLong™ Gold Antifade Mountant (Thermo Fisher Scientific Inc., Waltham, MA, USA). Sections were analyzed a laser scanning confocal microscope Leica TCS SP8 X (Leica Microsystems GmbH, Mannheim, Germany). Quantitative analysis of CD206 and IL-10 immunostaining was carried out using ImageJ 1.53 [32] evaluating the positive area on five randomly selected fields for each sample acquired with constant parameters.

### 2.6. In Vitro Studies

Healthy 8-week-old female athymic nude mice (Charles River Laboratories, Calco, Italy) were sacrificed by cervical dislocation. After sacrifice, peritoneal cells were obtained by peritoneal lavage as previously described with slight modifications [28]. The cells were seeded in 24-well plates (Corning, Glendale, AZ, USA) at a density of 5 × 10^5^ cells/well and incubated at 37 °C in 5% CO_2_ for 4 h. After incubation, the nonadherent cells were harvested and seeded in another 24-well plate (Corning, Glendale, AZ, USA). Fresh medium (RPMI 1640 medium supplemented with 10% FBS, 2 mM glutamine and 100 U/mL penicillin/streptomycin, all from Thermo Fisher Scientific Inc., Waltham, MA, USA) was added to the adherent and nonadherent cells. The peritoneal cells were then stimulated with 1 μM CpG-ODNs for 24 h. At the end of treatment, mRNA was extracted, and real-time PCR was performed to evaluate PD-1 expression.

RAW264.7 macrophages were seeded in 12-well plates (Corning, Glendale, AZ, USA) at a density of 8 × 10^5^ cells/well. The cells were then stimulated with 1 µM CpG-ODNs or 10 µg/mL anti-mouse PD-1 antibody, anti-mouse PD-1 antibody F(ab)_2_, anti-mouse PD-1 antibody Fc Silent™ or rat IgG2a isotype control antibody alone or in combination for 6 h. To block FcγR2b and FcγR3, RAW264.7 cells were pretreated for 30 min with 10, 100 or 500 µg/mL rat anti-mouse CD16/32 blocking antibody (clone 2.4G2, BioXCell, West Lebanon, NH, USA) and treated as described above. Controls were left untreated. At the end of treatment, mRNA extraction was performed. The time point was chosen based on our previous experiences (data not shown) and on a recently published work [33] that showed that 6 h is the optimal time for evaluating the expression of M1- and M2-related genes following macrophage stimulation.

### 2.7. Quantitative PCR Analysis

mRNA was extracted from RAW264.7 cells using Direct-zol™ RNA MicroPrep (Zymo Research, Irvine, CA, USA) according to the manufacturer’s instructions and reverse-transcribed with a High-Capacity RNA-to-cDNA Kit (Applied Biosystems-Thermo Fisher Scientific Inc., Waltham, MA, USA). Real-time PCR was performed using TaqMan^®^ Fast Universal PCR Master Mix (Applied Biosystems-Thermo Fisher Scientific Inc., Waltham, MA, USA) on a StepOne Real-Time PCR System (Applied Biosystems-Thermo Fisher Scientific Inc., Waltham, MA, USA). Table S1 lists all the TaqMan^®^ gene expression assays (Applied Biosystems-Thermo Fisher Scientific Inc., Waltham, MA, USA) utilized for real-time PCR analysis. The expression of each gene was normalized to β2m expression. PCR data were analyzed using the 2^−ΔCt^ method as previously described [34].

### 2.8. Generation of Bone Marrow-Derived Macrophages (BMDMs) and Isolation of NK Cells

To generate BMDMs, bone marrow cells were harvested from 8-week-old female healthy athymic nude mice (Charles River Laboratories, Calco, Italy), as previously described [35,36] with slight modifications. Briefly, bone marrow progenitors were harvested from the femur and tibia via fine dissection. Erythrocytes were lysed using ACK Lysing Buffer (Thermo Fisher Scientific Inc., Waltham, MA, USA) and bone marrow cells were cultured in Iscove’s Modified Dulbecco’s Medium (IMDM, Thermo Fisher Scientific Inc., Waltham, MA, USA) supplemented with 10% FBS (Thermo Fisher Scientific Inc., Waltham, MA, USA), 1% penicillin and streptomycin (Thermo Fisher Scientific Inc., Waltham, MA, USA) and 20 ng/mL macrophage-colony stimulating factor (M-CSF, PeproTech, Cranbury, NJ, USA) in 5% CO_2_ at 37 °C. On day 3, the culture medium was replaced with fresh medium containing M-CSF. BMDMs were harvested after 7 days of M-CSF–mediated macrophage differentiation. Untouched NK cells were isolated from the spleen of 8-week-old female healthy athymic nude mice (Charles River Laboratories, Calco, Italy) using NK Cell Isolation Kit, mouse (Miltenyi Biotec, Bergisch Gladbach, Germany) according to the manufacturer’s instructions.

### 2.9. ^51^Cr Release Assay

BMDMs were seeded in 24-well plates (Corning, Glendale, AZ, USA) at a density of 8 × 10^5^ cells/well. The cells were then stimulated with 1 µM CpG-ODNs, 10 µg/mL anti-mouse PD-1 antibody, 10 µg/mL anti-mouse PD-1 antibody Fc Silent™ alone or in combination for 6 h. At the end of treatment, the culture medium containing the different agents was removed and 8 × 10^5^ spleen-isolated NK cells were added to each experimental condition and co-cultured with BMDMs for 36 h. At the end of the co-incubation, NK cells were harvested and cytotoxicity assay was performed as previously described [34]. Briefly, YAC-1 cells were loaded with 100 μCi ^51^Cr (PerkinElmer, Waltham, MA, USA) for 1 h at 37 °C, washed 3 times with PBS containing 5% FBS, and resuspended in IMDM containing 10% FBS. NK cells were then co-incubated with YAC-1 target cells at 50:1 effector:target ratio in quadruplicate 96-well U-bottomed plates (Corning, Glendale, AZ, USA) for 4 h at 37 °C. The radioactivity in the supernatant (80 μL) was measured using a Trilux Beta scintillation counter (PerkinElmer, Waltham, MA, USA). Percentage of specific lysis was calculated as follows: ((experimental cpm − spontaneous cpm)/(maximum cpm − spontaneous cpm)) × 100. Cpm: counts per minute.

### 2.10. Statistical Analysis

The data are presented as the mean ± standard error (mean ± SEM). Statistical analysis was performed using GraphPad Prism (GraphPad Software, San Diego, CA, USA). Prior to each statistical analysis, the normal distribution of data was tested with normality tests. Comparisons between two experimental groups were performed using two-tailed unpaired Student’s *t*-test or the Mann–Whitney U test for data without normal distribution. Comparisons among three or more experimental groups were performed using one-way ANOVA followed by Tukey’s multiple comparison test with a single pooled variance or using the Kruskal–Wallis test followed by Dunn’s multiple comparison test for nonparametric data. Differences were considered significant at *p* < 0.05.

## 3. Results

### 3.1. TLR9 Stimulation by CpG-ODNs Enhances PD-1 Expression on Peritoneal Immune Cells

**Figure 1:**
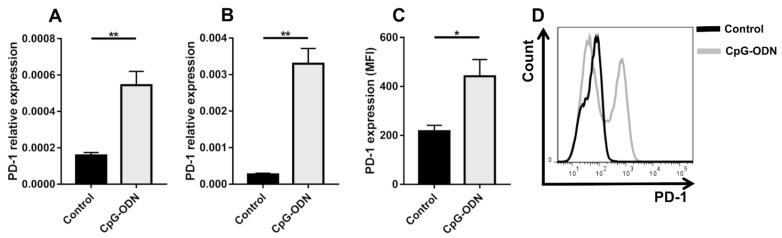
CpG-ODN treatment induces PD-1 expression on peritoneal immune cells in vitro and in vivo. PD-1 mRNA expression, as quantified by real-time PCR, in adherent (**A**) and nonadherent (**B**) peritoneal immune cells isolated from healthy mice and stimulated in vitro with 1 μM CpG-ODNs for 24 h. The data were normalized to the expression of β2m as a housekeeping reference gene and analyzed by the comparative 2^−ΔCt^ method. (**C**) Mean fluorescence intensity (MFI) and (**D**) a representative histogram plot of PD-1 expression on peritoneal CD45^+^ cells isolated from healthy nude mice treated i.p. with 20 μg/mouse CpG-ODNs or saline for 4 consecutive days. Black line: control; Gray line: CpG-ODN. The data are presented as the mean ± SEM. * *p* < 0.05 and ** *p* < 0.01 by two-tailed unpaired Student’s *t*-test. CpG-ODNs: CpG oligodeoxynucleotides.

It has been reported that TLR9 stimulation leads to upregulation of PD-1 expression on NK cells [11], monocytes [12] and B lymphocytes [13]. In line with these data, in vitro TLR9 stimulation was able to increase mRNA PD-1 expression on the adherent and nonadherent fractions of peritoneal immune cells isolated from healthy athymic nude mice, which were mostly macrophages and B lymphocytes, respectively [28] (Figure 1A,B). Accordingly, flow cytometry analysis of peritoneal immune infiltrates obtained from peritoneal lavage revealed augmented PD-1 expression on CD45^+^ cells in athymic mice treated i.p. for 4 days with CpG-ODNs compared to untreated mice (Figure 1C,D).

These findings indicate that TLR9-induced upregulation of PD-1 expression, probably as a part of a complex negative feedback mechanism, may limit the antitumor activity of CpG-ODNs and provide a rationale for combining TLR9 agonism and PD-1 blockade to reverse innate immune system exhaustion.

### 3.2. An Anti-PD-1 Antibody Reduces the Antitumor Activity of CpG-ODNs in Athymic Nude Mice Bearing Human Ovarian Cancer Xenografts

**Figure 2:**
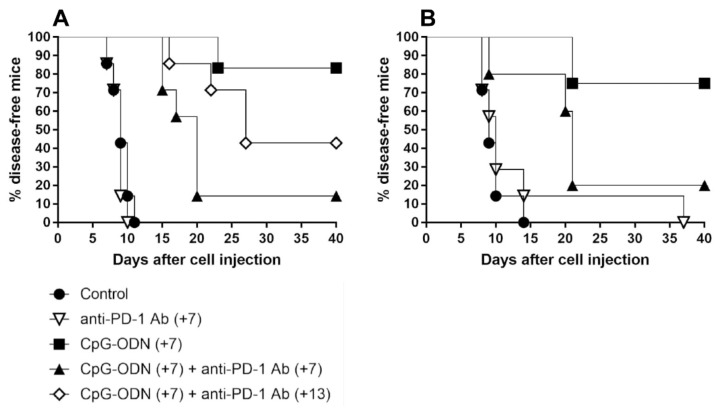
Kaplan–Meier plot of percent disease-free IGROV-1 ovarian tumor-bearing mice treated with CpG-ODNs, an anti-PD-1 antibody or their combination. (**A**) Mice xenografted with IGROV-1 human ovarian cancer cells were treated i.p. with CpG-ODNs (20 μg/mouse, 5 days/week for 3 weeks, starting seven days after tumor cell injection), treated i.p. with an anti-PD-1 antibody (clone RMP1-24) (200 μg/mouse, 2 times/week for 3 weeks, starting seven (+7) or thirteen (+13) days after tumor cell injection) or administered the combination of these treatments. (**B**) Mice xenografted with IGROV-1 human ovarian cancer cells were treated i.p. with CpG-ODNs (20 μg/mouse, 5 days/week for 3 weeks, starting seven days after tumor cell injection), treated i.p. with an anti-PD-1 antibody (clone J43) (200 μg/mouse, 2 times/week for 3 weeks, starting seven (+7) days after tumor cell injection) or administered the combination of these treatments. The control group received saline. Numbers in parentheses indicate the number of days between tumor cell injection and the administration of the first treatment. CpG-ODNs: CpG oligodeoxynucleotides; anti-PD-1 Ab: anti-PD-1 antibody.

Athymic immunodeficient mice were i.p. injected with IGROV-1 human ovarian carcinoma cells and treated with CpG-ODNs alone or in combination with an anti-mouse PD-1 antibody seven days after tumor cell injection. As expected [6,7,8], ascites appearance was markedly delayed in CpG-ODN-treated mice compared to controls and mice treated with the anti-PD-1 antibody, which, when administered alone, did not show antitumor effects (control group vs. CpG-ODN-treated group, *p* = 0.0004 by the log-rank test; anti-PD-1 antibody-treated group vs. CpG-ODN-treated group, *p* = 0.0005 by the log-rank test). Unexpectedly, there was a drastic reduction in the percentage of disease-free mice in the experimental group treated concomitantly with the anti-PD-1 antibody and TLR9 agonist compared to the group treated with CpG-ODN alone (CpG-ODN-treated group vs. CpG-ODN/anti-PD-1 antibody-treated group, *p* = 0.0079 by the log-rank test). A similar trend was also noticed when anti-PD-1 antibody treatment was started one week after the initiation of CpG-ODN administration (Figure 2A and Table S2) and in an early IGROV-1 ovarian cancer model in which mice received the first dose of CpG-ODNs and the anti-PD-1 antibody 5 and 8 days after tumor cell injection, respectively (data not shown). Another anti-mouse PD-1 blocking antibody (clone J43), which recognizes a different epitope of the PD-1 receptor than clone RMP1-14 [37], exerted comparable effects (Figure 2B and Table S2), indicating that the detrimental effect associated with the combinatorial treatment was not related to the anti-PD-1 antibody clone utilized.

Moreover, to investigate whether the same phenomenon can also occur in immunocompetent mice, in vivo experiments were carried out using mice bearing two poorly immunogenic tumors in which PD-1 blockade has been reported to not exert any therapeutic effect [38,39], as observed in the IGROV-1 model (Figure 2A,B). To this aim, mammary tumor 4T1 and melanoma B16 cells were s.c. injected into BALB/c and C57BL/6 mice, respectively, and the mice were treated p.t. with CpG-ODN alone or in combination with the anti-PD-1 antibody. As shown in Figure S1A,B, CpG-ODN activity was not affected by anti-PD-1 antibody coadministration.

Collectively, these data suggest that upon interaction with an anti-PD-1 antibody, innate immune cells are able to impair the therapeutic efficacy of TLR9 agonists, compromising the benefit of such a combinatorial treatment.

### 3.3. Macrophages Are the Innate Immune Cells Responsible for the Impairment of the Antitumor Activity of CpG-ODNs

**Figure 3:**
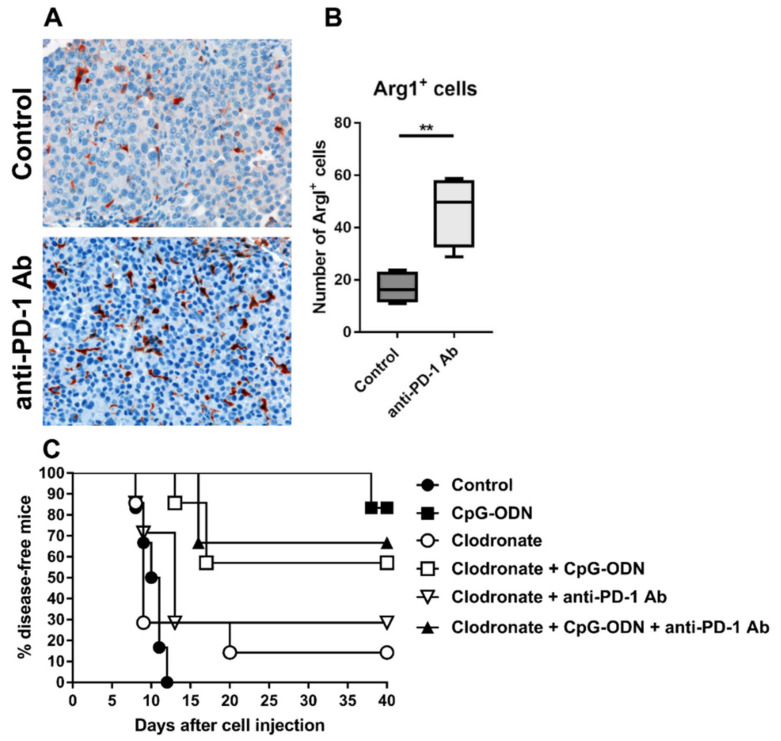
Role of macrophages in the dampening of the antitumor activity of CpG-ODNs. (**A**,**B**) An anti-mouse PD-1 antibody induced the accumulation of arginase I+ (ArgI^+^) myeloid elements in the tumor microenvironment. Eight-week-old female athymic nude mice were i.p. injected with IGROV-1 cells and treated i.p. with a monoclonal anti-mouse PD-1 antibody at a dose of 200 µg/mouse 5 times at two- or three-day intervals. Treatment started the day after tumor cell injection. Control mice received saline. (**A**) Representative histopathological evaluation and (**B**) quantification of ArgI-stained IGROV-1 tumor samples from mice treated with the anti-PD-1 antibody or untreated mice. Original magnification: 20 X. (**C**) Kaplan–Meier plot of percent disease-free IGROV-1 ovarian tumor-bearing mice treated with CpG-ODNs, an anti-PD-1 antibody or their combination after macrophage depletion. Mice xenografted with IGROV-1 human ovarian cancer cells were treated i.p. with liposomes containing clodronate three days after tumor cell injection. The animals were then i.p. injected with CpG-ODN (20 μg/mouse, 5 days/week for 3 weeks, starting seven days after tumor cell injection), i.p. injected with an anti-PD-1 antibody (clone RMP1-24) (200 μg/mouse, 2 times/week for 3 weeks, starting seven days after tumor cell injection) or administered the combination of these treatments. The control group received saline. ** *p* < 0.01 by unpaired *t*-test. CpG-ODNs: CpG oligodeoxynucleotides; anti-PD-1 Ab: anti-PD-1 antibody.

PD-1 blockade can sustain immunosuppression and protumor effects in ovarian and lung cancer upon interaction with myeloid/macrophage cells [26,40]. For example, anti-PD-1 antibody administration has been reported to promote the infiltration of ArgI^+^ M2-like macrophages in non-small cell lung cancer preclinical models [26]. Accordingly, immunohistochemical analysis of IGROV-1 tumor specimens revealed a significantly increased number of ArgI^+^ cells in anti-PD-1 antibody-treated tumors than in control tumors (Figure 3A,B).

On the same line, immunofluorescence quantitative analysis showed increased infiltration of elements positive for CD206, a mannose receptor expressed by macrophages with M2 phenotype that are associated with tumor progression in ovarian cancer [41], in the tumor stroma of anti-PD-1-treated mice compared to the untreated group (Figure S2A,B). The augmented infiltration of macrophages in anti-PD-1 treated animals was also paralleled by a higher presence of cells positive for IL-10, an immunoregulatory cytokine that can be also expressed by M2 macrophages [42], compared to control mice (Figure S2C,D). These observations suggest that macrophages may be involved in restraining the antitumor efficacy of CpG-ODNs.

To ascertain the role of macrophages in regulating the decrease in the antitumor activity of CpG-ODNs upon anti-PD-1 antibody delivery, macrophages were depleted in IGROV-1 tumor-bearing mice by in vivo administration of liposomes containing clodronate and, the mice were subsequently treated as described above. As shown in Figure 3C, the absence of macrophages slightly affected the antitumor activity of CpG-ODNs, but the effect was not significant (CpG-ODN-treated group vs. clodronate + CpG-ODN-treated group, *p* = 0.2743 by the log-rank test), indicating that these innate immune cells are partially involved in the antitumor activity of CpG-ODNs. Moreover, macrophage depletion completely abrogated the inhibitory effect of the anti-PD-1 antibody on the antitumor effect of CpG-ODN, suggesting that macrophages may drive the dampening of the therapeutic efficacy of CpG-ODNs. No differences were found between the macrophage-depleted anti-PD-1 antibody-treated group and control mice, as observed in Figure 2A,B (Figure 3C).

### 3.4. Effect of the Interaction between the Anti-PD-1 Antibody Fc Domain and Macrophages on the Antitumor Efficacy of CpG-ODNs

**Figure 4:**
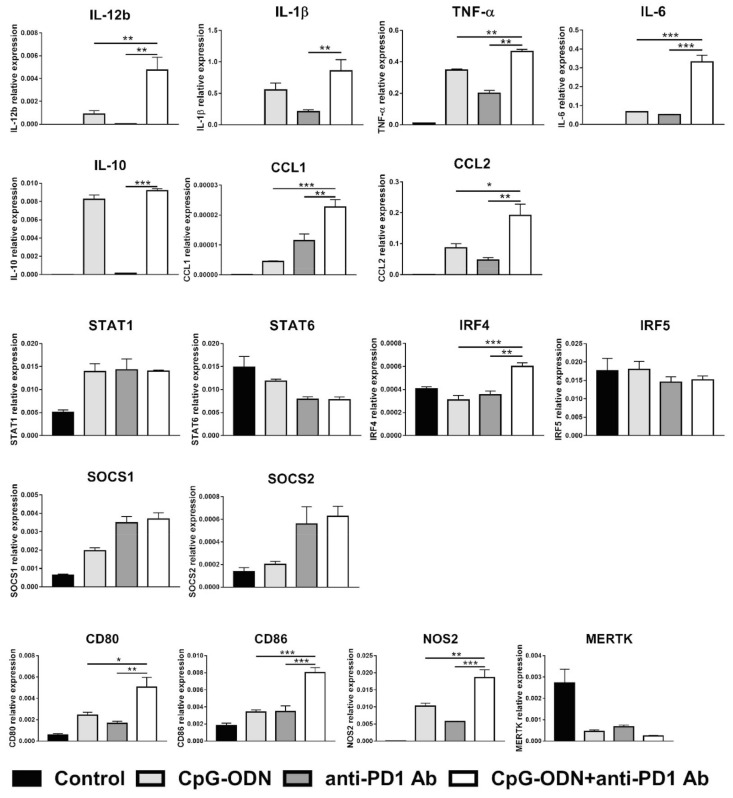
Effect of the combination of CpG-ODNs and an anti-mouse PD-1 antibody on RAW264.7 macrophages in vitro. RAW264.7 macrophages were seeded in 12-well plates and then stimulated with 1 µM CpG-ODNs and 10 µg/mL anti-mouse PD-1 antibody alone or in combination for 6 h. Controls were left untreated. At the end of treatment, mRNA was extracted, and real-time PCR was performed. The data were normalized to the expression of β2m as a housekeeping reference gene and analyzed by the comparative 2^−ΔCt^ method. The data are presented as the mean ± SEM. * *p* < 0.05, ** *p* < 0.01, and *** *p* < 0.001 by one-way ANOVA followed by Tukey’s multiple comparison test with a single pooled variance or by the Kruskal–Wallis test followed by Dunn’s multiple comparison test for nonparametric data. CpG-ODNs: CpG oligodeoxynucleotides; anti-PD-1 Ab: anti-PD-1 antibody.

The effect of the combination of CpG-ODNs and an anti-PD-1 antibody (rat IgG2a anti-mouse PD-1 clone RMP1-14) on macrophages was evaluated in vitro by exposing RAW264.7 mouse macrophages to each reagent alone or in combination for 6 h. At the end of treatment, real-time PCR was performed to evaluate the expression of M1- and M2-macrophage-specific markers as previously described [43,44]. IL-12b is considered a hallmark of M1 macrophages, while all the other markers analyzed are expressed by both M1 and M2 macrophages [43,44]. Figure 4 shows that the expression of the cytokines IL-12b, TNF-α, and IL-6 and the chemokines CCL1 and CCL2 was significantly upregulated in the combinatorial treatment group compared to the single treatment groups.

Moreover, the expression of the activation markers CD80 and CD86 was more strongly increased in macrophages by CpG-ODN/anti-PD-1 antibody double treatment than by each reagent alone not only at mRNA but also at the protein level, as evaluated by flow cytometry analysis (Figure S3A,B). Similar findings were observed for NOS2, a gene encoding a protein involved in amino acid metabolism [45]. Interestingly, when the expression of different transcription factors important in mediating the acquisition of the M1 or M2 phenotype was evaluated, a higher level of IRF4 mRNA, a peculiar M2 transcription factor [46], was observed when CpG-ODNs and the anti-PD-1 antibody were co-administered than when either reagent was administered alone (Figure 4).

To further ascertain whether the combinatorial regimen could favor a polarization reprogramming toward an M2 phenotype, RAW264.7 cells were treated as above and, subsequently, stained for CD206. We found that this marker is expressed at a very low level in macrophages left untreated or treated with CpG-ODN or anti-PD-1 antibody alone. Notably, the combinatorial group was characterized by a slight but appreciable increased CD206 expression compared to the other experimental conditions (Figure S4A). Accordingly, athymic mice injected with IGROV-1 cells showed an increased infiltration of CD206^+^ macrophages when treated with CpG-ODN in combination with anti-PD-1 antibody compared to CpG-ODN alone (Figure S4B,C). Moreover, immunofluorescence analysis also revealed an augmented IL-10 signal in CpG-ODN/anti-PD-1-treated tumors compared to the CpG-ODN-treated group (Figure S4D,E).

Since macrophages and RAW264.7 cells express PD-1 [47] (Figure S5), whose expression can be modulated by TLR9 ligation [12] (Figure S5), we investigated whether the changes in gene expression observed in RAW264.7 cells treated with the combination of CpG-ODNs and the anti-PD-1 antibody could be due to direct PD-1 stimulation. To this end, RAW264.7 cells were treated with the anti-PD-1 antibody F(ab)_2_, which lacks an Fc domain but retains the ability to bind PD-1 [26] (Figure S6).

**Figure 5:**
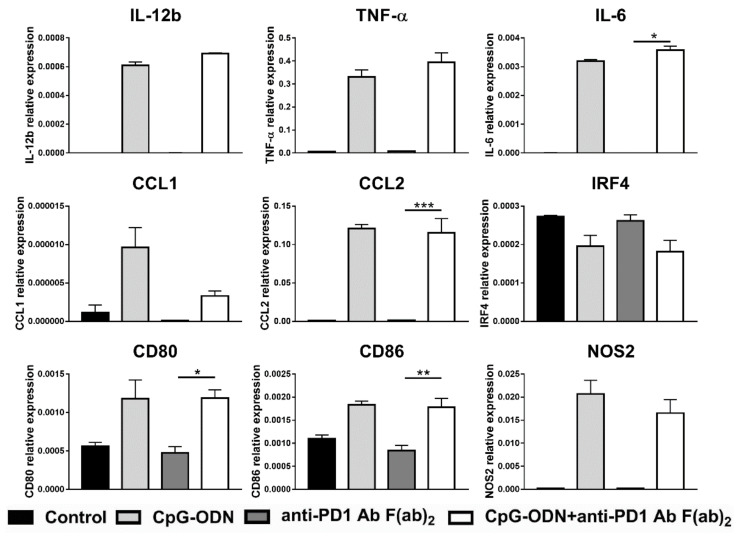
Effect of the combination of CpG-ODNs and anti-mouse PD-1 antibody F(ab)_2_ on RAW264.7 macrophages in vitro. RAW264.7 macrophages were seeded in 12-well plates and then stimulated with 1 µM CpG-ODN and 10 µg/mL anti-mouse PD-1 antibody F(ab)_2_ alone or in combination for 6 h. Controls were left untreated. At the end of treatment, mRNA was extracted, and real-time PCR was performed. The data were normalized to the expression of β2m as a housekeeping reference gene and analyzed by the comparative 2^−ΔCt^ method. The data are presented as the mean ± SEM and were analyzed by one-way ANOVA followed by Tukey’s multiple comparison test with a single pooled variance or by the Kruskal–Wallis test followed by Dunn’s multiple comparison test for nonparametric data. * *p* < 0.05, ** *p* < 0.01, and *** *p* < 0.001. CpG-ODNs: CpG oligodeoxynucleotides; anti-PD-1 Ab: anti-PD-1 antibody.

Genes found to be significantly differently expressed in the combinatorial treatment group compared to the single agent-treated groups (see Figure 4) were considered for the analysis. All the considered genes showed no differences in mRNA expression levels between CpG-ODN/anti-PD-1 antibody F(ab)_2_- and CpG-ODN-treated macrophages, indicating that PD-1 receptor blockade does not synergize with CpG-ODNs to exert the same biological effect as the whole antibody on macrophages (Figure 5).

**Figure 6:**
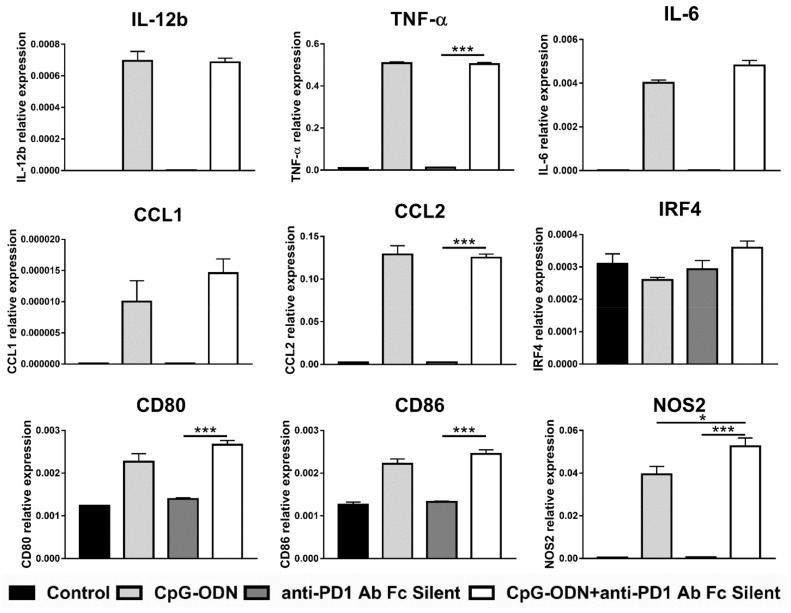
Effect of the combination of CpG-ODNs and the Fc silent anti-mouse PD-1 antibody on RAW264.7 macrophages in vitro. RAW264.7 macrophages were seeded in 12-well plates and then stimulated with 1 µM CpG-ODN and 10 µg/mL Fc silent anti-mouse PD-1 antibody alone or in combination for 6 h. Controls were left untreated. At the end of treatment, mRNA was extracted, and real-time PCR was performed. The data were normalized to the expression of β2m as a housekeeping reference gene and analyzed by the comparative 2^−ΔCt^ method. The data are presented as the mean ± SEM and were analyzed by one-way ANOVA followed by Tukey’s multiple comparison test with a single pooled variance or by the Kruskal–Wallis test followed by Dunn’s multiple comparison test for nonparametric data. * *p* < 0.05 and *** *p* < 0.001. CpG-ODNs: CpG oligodeoxynucleotides; anti-PD-1 Ab: anti-PD-1 antibody.

To corroborate the results obtained with the anti-PD-1 antibody F(ab)_2_, macrophages were incubated with an engineered anti-PD-1 antibody-containing key point mutations in its Fc domain that abrogate its binding to Fc receptors, named anti-PD-1 antibody Fc silent. In line with the previous experiment, none of the analyzed genes were found differentially modulated between CpG-ODN/anti-PD-1 antibody Fc silent- and CpG-ODN-treated macrophages, with the exception of NOS2, the expression of which was significantly but very modestly upregulated in the CpG-ODN/anti-PD-1 antibody-treated group compared to the CpG-ODN alone-treated group (Figure 6).

**Figure 7:**
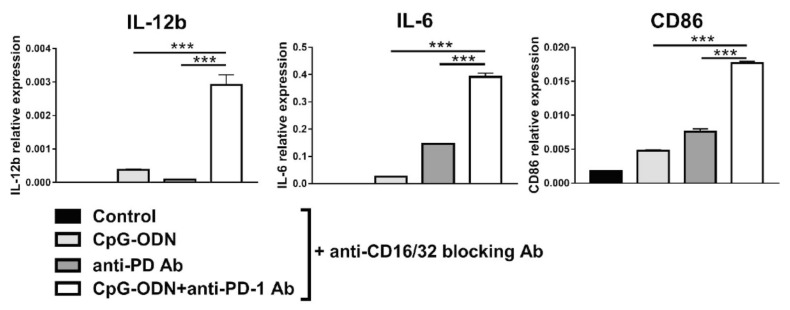
Influence of blocking FcγR2b (CD32) and FcγR3 (CD16) on the effect of the combination of CpG-ODN and an anti-mouse PD-1 antibody on RAW264.7 macrophages in vitro. RAW264.7 macrophages were seeded in 12-well plates and then pretreated for 30 min with 10 µg/mL rat anti-mouse CD16/32 blocking antibody. Subsequently, the cells were stimulated with 1 µM CpG-ODN and 10 µg/mL anti-mouse PD-1 antibody alone or in combination for 6 h. Controls were left untreated. At the end of treatment, mRNA was extracted, and real-time PCR was performed. The data were normalized to the expression of β2m as a housekeeping reference gene and analyzed by the comparative 2^−ΔCt^ method. The data are presented as the mean ± SEM. *** *p* < 0.001 by one-way ANOVA followed by Tukey’s multiple comparison test with a single pooled variance or by the Kruskal–Wallis test followed by Dunn’s multiple comparison test for nonparametric data. CpG-ODNs: CpG oligodeoxynucleotides; anti-PD-1 Ab: anti-PD-1 antibody.

These results tend to exclude the possibility that PD-1 blockade alone is responsible for the observed changes in gene expression in macrophages, indicating a role for the anti-PD-1 antibody Fc domain in this phenomenon, as previously hypothesized [26,48]. Blocking FcγR2b and FcγR3, which reportedly interact with the anti-PD-1 antibody Fc domain [49,50], did not reverse the effect of the combination of the anti-PD-1 antibody and CpG-ODNs on macrophage marker expression profiles (Figure 7), probably because the upregulation of the mRNA expression of these Fc receptors induced by the combinatorial treatment prevented the blockade of all FcγR2b and FcγR3 expressed on the macrophage cell membrane (Figure S7).

**Figure 8:**
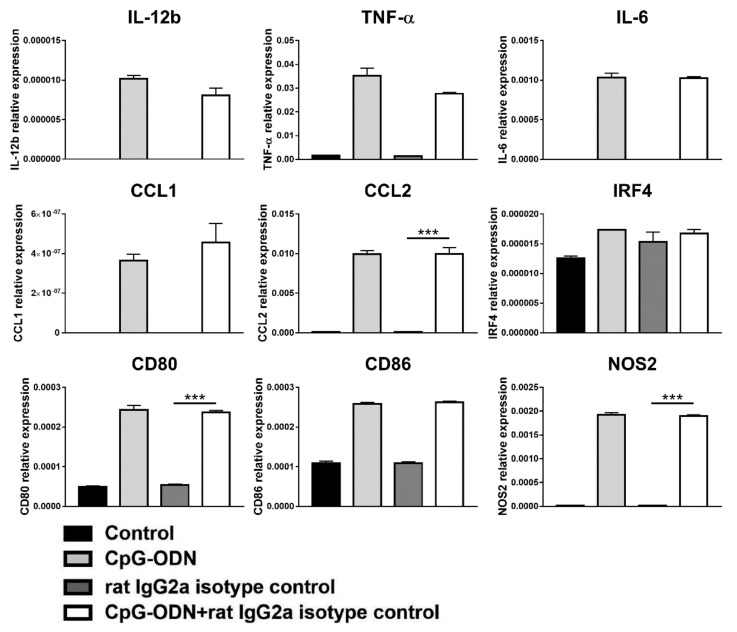
Effect of the combination of CpG-ODNs and rat IgG2a isotype control antibody on RAW264.7 macrophages in vitro. RAW264.7 macrophages were seeded in 12-well plates and then stimulated with 1 µM CpG-ODN and 10 µg/mL rat IgG2a isotype control antibody alone or in combination for 6 h. Controls were left untreated. At the end of treatment, mRNA was extracted, and real-time PCR was performed. The data were normalized to the expression of β2m as a housekeeping reference gene and analyzed by the comparative 2^−ΔCt^ method. The data are presented as the mean ± SEM and were analyzed by one-way ANOVA followed by Tukey’s multiple comparison test with a single pooled variance or by the Kruskal–Wallis test followed by Dunn’s multiple comparison test for nonparametric data. *** *p* < 0.001. CpG-ODNs: CpG oligodeoxynucleotides; anti-PD-1 Ab: anti-PD-1 antibody.

In addition, exposure of macrophages to CpG-ODNs in combination with a rat IgG2a isotype-matched control antibody did not exert any effect on the expression of the analyzed genes (Figure 8), unlike clone RMP1-14 (Figure 4).

Finally, it has been investigated whether the changes in gene expression also have functional repercussions in macrophages. Since it has been reported that macrophages can profoundly impact NK cell cytotoxicity [34,51], the ability of these immune cells to modulate NK cytotoxic activity after exposure to CpG-ODN, anti-PD-1 antibody or their combination, as a mirror of the functional features acquired by macrophage upon different treatments, has been evaluated. As expected, CpG-ODN-stimulated BMDMs were able to boost NK cell cytotoxicity (Figure S8). Similar results were obtained when BMDMs were treated with anti-PD-1 antibody. Notably, NK cells incubated with BMDMs previously exposed to CpG-ODN/anti-PD-1 antibody combination showed a cytotoxic activity comparable to those co-cultured with untreated BMDMs. It was also observed that the anti-PD-1 antibody Fc silent did not prime macrophages to induce NK cytotoxic activity, as observed with the anti-PD-1 antibody. In CpG-ODN/ anti-PD-1 antibody Fc silent treated BMDMs, the lysis exerted by NK cells can be only ascribed to CpG-ODN immunostimulatory capability (Figure S8). These data support the idea that the Fc domain is important in mediating macrophage reprogramming after TLR-stimulation.

Collectively, these findings indicate a scenario in which the Fc domain of the anti-PD-1 antibody stimulates a signaling cascade in macrophages, regulating their polarization. However, previous binding of an anti-PD-1 antibody to its specific target, PD-1, on macrophages is an essential prerequisite for the Fc domain to exert its biological function.

In conclusion, our data demonstrated that antigen binding and the Fc domain of the anti-PD-1 antibody are both necessary for the modification of macrophage phenotypes by the antibody in combination with a TLR9 agonist.

## 4. Discussion

In the present study, we observed that CpG-ODN stimulation led to PD-1 expression upregulation in peritoneal immune cells in vitro and in vivo. Our data are in line with other reports showing that PD-1 expression can be increased by TLR9 ligands in NK cells [11], monocytes/macrophages [12] and B lymphocytes [13]. PD-1 on T lymphocytes and innate immune cells is related to effector function impairment and reduced immunoresponsiveness. Therefore, blockade of PD-1 signaling may be a successful strategy for improving the immunostimulatory effects of TLR agonists, as supported by several preclinical studies in immunocompetent mice [22,23,24,38].

We attempted to unravel the contribution of the innate immune system to the effect of combinatorial immunotherapeutic treatments based on TLR9 stimulation and PD-1 blockade in an IGROV-1 immunodeficient mouse model and observed a strong reduction in the antitumor efficacy of a TLR9 agonist upon anti-PD-1 antibody administration. These results indicate that innate immune cells exposed to the combination of these two immunotherapeutic drugs can acquire an immunosuppressive and protumor phenotype that can counteract the immunostimulatory function of CpG-ODNs. Evaluation of intratumor immune cell infiltrates revealed accumulation of macrophages with an M2-like phenotype in anti-PD-1 antibody-treated tumors, suggesting the involvement of these immune cells in determining the negative effect associated with the anti-PD-1 antibody. Accordingly, our data also showed that the detrimental effect exerted by the anti-PD-1 antibody on the antitumor activity of CpG-ODNs was abrogated in the absence of macrophages.

The role of macrophages in the effect of anti-PD-1 therapy has not yet been completely elucidated. Some authors have reported that blocking PD-1 on macrophages can restore the anticancer potential of these cells, promoting a shift from a protumor to an antitumor phenotype [47], while other studies have suggested that binding of an anti-PD-1 antibody to macrophages may exacerbate immunosuppression and thus impede successful immune checkpoint blockade therapy [12,26,52].

Real-time PCR analysis revealed that RAW264.7 macrophages treated with CpG-ODNs and an anti-PD-1 antibody exhibited a statistically significant increase in the mRNA expression of cytokines (TNF-α, IL-6), chemokines (CCL1 and CCL2) and activation markers (CD80 and CD86), which suggests the acquisition of the M2b phenotype, an immunoregulatory phenotype [44]. This particular M2 macrophage subtype, which was reported to be generated by the concomitant activation of TLRs and Fc receptors and involved in immunosuppression, cancer progression and resistance to therapy [53,54], expresses various genes [44], specifically those with expression levels that were upregulated by the combination of CpG-ODNs and the anti-PD-1 antibody in our cellular model. Accordingly, the expression of chemokine CCL1, which is considered a specific marker of M2b macrophages [44], was markedly increased in the combinatorial treatment group compared to single-agent-treated groups. Furthermore, the expression of IRF4, an M2 marker gene transcription factor [46], was found to be upregulated in the combinatorial treatment group, favoring the acquisition of the M2 immunosuppressive phenotype. Of note, the increase in the expression of IL-6, another M2b marker [44] that can promote tumor growth and ascites formation in ovarian cancer [55,56], may explain the acceleration of ascites development in CpG-ODN/anti-PD-1 antibody-treated mice.

Our data also revealed mRNA overexpression of IL-12b, an M1-related cytokine [43], in the CpG-ODN/anti-PD-1 antibody-treated group. Although this finding may be inconsistent with the acquisition of an M2b phenotype, it is possible that the combination of TLR9 stimulation and PD-1 blockade may not produce “canonical” M2b macrophages but instead macrophages with a mixed phenotype that share the characteristics of M1 and M2 macrophages. On the other hand, it has been demonstrated that IL-10, the expression of which was induced by CpG-ODN, can inhibit IL-12b production by inducing epigenetic modification of the IL-12b promoter [57], probably interfering with IL-12 synthesis. Therefore, upregulation of IL-12b mRNA expression may not be indicative of the acquisition of the M1 phenotype. Regarding IL-10, we observed an increased infiltration of IL-10^+^ elements either in anti-PD-1-treated IGROV-1 tumors compared to controls (Figure S2), and in CpG-ODN/anti-PD-1 antibody group compared to mice injected with CpG-ODN alone (Figure S4). However, in vitro studies indicated that this cytokine was up-regulated in macrophages only by TLR9 stimulation and that anti-PD-1 antibody did not influence IL-10 expression (Figure 4). Considering that it has already been demonstrated that PD-1 blockade induced IL-10 secretion by myeloid cells in ovarian cancer [40], these apparent discrepancies could be explained by the fact that six hours of in vitro treatment were not sufficient to allow IL-10 modulation by the anti-PD-1 antibody.

Collectively, the in vitro data suggest that the combinatorial treatment can polarize macrophages towards an M2b-like phenotype, an immunosuppressive and protumor subphenotype that can restrain the antitumor activity of CpG-ODNs. The that macrophages acquire an M2b phenotype may also be supported by the in vitro data indicating a role for macrophage Fc receptors that, when triggered by the anti-PD-1 antibody Fc domain, polarize macrophages toward this particular phenotype in synergy with a TLR9 agonist.

Indeed, the results of the experiments performed using either anti-PD-1 antibody F(ab)_2_ or an anti-PD-1 antibody with no ability to bind any Fc receptors not only support the idea that PD-1 blockade alone has a negligible effect on macrophage behavior but also suggest that the Fc region of the anti-PD-1 antibody may be responsible for the observed changes in macrophage-related gene expression upon TLR9 stimulation. However, treatment of macrophages with a rat IgG2a isotype control, which possesses the same Fc region as the anti-PD-1 antibody clone RMP1-14, and CpG-ODNs did not exert any biological effect on macrophages. Collectively, these findings may indicate that, although it is not sufficient to directly polarize macrophages, binding of the antibody to PD-1 might be necessary for the anti-PD-1 antibody Fc domain to adopt the correct conformation, allowing it to interact with the Fc receptors expressed by macrophages on the same cell or on adjacent cells [58]. However, we cannot exclude the possibility that simultaneous triggering of the signaling pathways downstream of PD-1 and the Fc receptors induced by the anti-PD-1 antibody may be needed in combination with TLR9 stimulation to shape the macrophage transcriptome.

As previously mentioned, the anti-PD-1 antibody Fc domain (clone RMP1-14) can only interact with mouse FcγR2b (CD32b) and FcγR3 (CD16) [49,50]. However, blocking these receptors with increasing concentrations of a specific anti-CD16/32 antibody (ranging from 10 to 500 μg/mL, Figure 7 and data not shown) did not abrogate or mitigate the effect of the CpG-ODN/anti-PD-1 antibody combination on macrophages. Although these data appear difficult to explain, it is possible that the anti-CD16/32 antibody did not block all the Fc receptors expressed on the macrophage cell membrane. In line with this view, we found that the CpG-ODN/anti-PD-1 antibody combination was responsible for the upregulation of the mRNA expression of FcγR2b and FcγR3 in RAW264.7 cells (Figure S7), supporting the possibility that there were remaining Fc receptors to interact with anti-PD-1 antibody after anti-CD16/32 antibody pretreatment. Although this scenario seems plausible, in our opinion, we cannot fully explain why we did not observe at least a reduction in the effect of the combination of the anti-PD-1 antibody and TLR9 stimulation upon FcγR2b/FcγR3 blockade. Indeed, a preliminary experiment performed using decreasing concentrations (from 10 μg/mL to 0.313 μg/mL) of anti-PD-1 antibody in combination with CpG-ODNs clearly demonstrated that the effect of the anti-PD-1 antibody on macrophages is dose-dependent (Figure S9), suggesting that the number of Fc receptors interacting with the anti-PD-1 antibody correlates with the magnitude of the effect on macrophages.

Therefore, this finding is not perfectly consistent with the notion that only a small amount of Fc receptors may be necessary for the effect of the CpG-ODN/anti-PD-1 antibody combination. For this reason, we hypothesize that other receptors may interact with the anti-PD-1 antibody Fc domain and play a crucial role in mediating the observed phenomenon. For instance, it has been reported that macrophages can express a broad repertoire of receptors for immunoglobulins [59], the cognate ligands of most of which are still poorly characterized. Further studies are needed to fully identify the Fc receptor(s) involved in the observed effect on macrophages.

## 5. Conclusions

We are aware that our immunodeficient preclinical model does not recapitulate the situation in humans. Moreover, only a few clinical trials have investigated the combination tested in this study [4], suggesting that only a small fraction of patients may suffer adverse effects from this type of combinatorial regimen. However, it should be highlighted that the data shown in the present manuscript may be translated into broader and more complicated biological contexts. Indeed, TLR signaling activation can be achieved not only by synthetic agonists but also by a plethora of molecules present in the tumor microenvironment. For example, components of the extracellular matrix, DNA released by apoptotic cells, and heat-shock proteins are potential TLR agonists, the so-called DAMPs [60]. In addition, tumor-associated microbiota [61,62] or bacteria colonizing different tissues [62,63,64] may be continuous sources of microbial products that can stimulate TLRs in immune cells, as described in the peritoneum [65], a common site of metastasis of ovarian cancer [66]. It is possible that even when delivered as a monotherapy, anti-PD-1 antibodies may act on macrophages in which TLR signaling has already been triggered by endogenous ligands, mirroring the biological effects described in the present study. This hypothesis might also provide a mechanistic explanation of the clinical phenomenon of hyperprogressive disease, a peculiar pattern of progression observed in patients receiving anti-PD-1 antibodies characterized by an exacerbation of tumor growth and metastatic potential, which has been proposed to be mediated by tumor-associated macrophages [48]. Therefore, if a tumor mass is abundantly infiltrated by macrophages or if it is growing in an anatomical site particularly enriched in resident macrophages, it is possible that anti-PD-1 antibodies might generate macrophages with immunoregulatory activity that are able to abolish or dampen the efficacy of anticancer therapies and eventually promote tumor progression.

## Supplementary Materials

The following are available online at https://www.mdpi.com/article/10.3390/cancers13164081/s1, Figure S1: effect of the combination of TLR9 stimulation and PD-1 blockade in immunocompetent mice, Figure S2: immunofluorescence analysis of CD206^+^ and IL-10^+^ cells in IGROV-1 ovarian tumor after anti-PD-1 treatment, Figure S3: effect of the in vitro combination of CpG-ODNs and an anti-mouse PD-1 on CD80 and CD86 expression by RAW264.7 cells, Figure S4: effect of the combination of CpG-ODNs and an anti-mouse PD-1 antibody on macrophages in vitro and in vivo, Figure S5: PD-1 expression on RAW264.7 cells and its modulation by TLR9 stimulation, Figure S6: anti-PD-1 antibody F(ab)_2_ retains the ability to bind PD-1, Figure S7: modulation of FcγR2b and FcγR3 expression on RAW264.7 macrophages in vitro by the combination of CpG-ODNs and an anti-mouse PD-1 antibody, Figure S8: evaluation of the ability of in vitro stimulated-BMDMs to modulate NK cell cytotoxic activity, Figure S9: effect of CpG-ODNs in combination with different concentrations of anti-mouse PD-1 antibody on RAW264.7 macrophages in vitro. Table S1: List of TaqMan^®^ gene expression assays utilized for Real Time PCR analysis, Table S2: effect of CpG-ODN and anti-PD-1 antibody combination on ascites appearance in IGROV-1 tumor-bearing mice, Supplementary Figure Legends.
