# Supplementary material for: Macrophages Impair TLR9 Agonist Antitumor Activity through Interacting with the Anti-PD-1 Antibody Fc Domain
Source: Cancers (Basel). 2021 Aug 13;13(16):4081. doi: 10.3390/cancers13164081 (PMC8391891; doi:10.3390/cancers13164081)
Supplement: Supplementary file 1 [file cancers-13-04081-s001.zip › Supplementary Figure Legends Revised.pdf]

SUPPLEMENTARY FIGURE LEGENDS

Figure S1. Effect of the combination of TLR9 stimulation and PD-1 blockade in immunocompetent mice. (**A**) Balb/c mice xenografted s.c. with 4T1 breast carcinoma cells and p.t. treated with CpG-ODN alone (starting on day 10; 20 μg/mouse, 5 days/week) or with an anti-PD-1 antibody (200 μg/mouse, 2 days/week) starting on day 7 after tumor cell injection. The control group was left untreated. (**B**) C57BL/6 mice xenografted s.c. with B16 melanoma cells and treated with CpG-ODNs (20 μg/mouse, 5 days/week) alone or in combination with an anti-PD-1 antibody (200 μg/mouse, 2 days/week) starting 21 days after tumor cell injection. The control group was left untreated. The dots represent the mean±SEM of tumor volume for the different groups. **CpG-ODNs**: CpG oligodeoxynucleotides; **anti-PD-1 Ab**: anti-PD-1 antibody.

Figure S2. Immunofluorescence analysis of $CD206^+$ and $IL\text{-}10^+$ cells in IGROV-1 ovarian tumor after anti-PD-1 treatment. An anti-mouse PD-1 antibody induced the accumulation of $CD206^+$ and $IL\text{-}10^+$ cells in the tumor microenvironment. Eight-week-old female athymic nude mice were i.p. injected with IGROV-1 cells and treated i.p. with a monoclonal anti-mouse PD-1 antibody at a dose of 200 μg/mouse 5 times at two- or three-day intervals. Treatment started the day after tumor cell injection. Control mice received saline. (**A**) Representative immunofluorescence image of IGROV-1 tumor samples from mice treated with the anti-PD-1 antibody or left untreated stained for CD206 (green) and counterstained with 4′,6-diamidino-2-phenylindole (DAPI; blue) for nuclei. (**B**) Quantification of CD206 positive area. Box and whiskers: min to max of data analyzing the positive area of five randomly selected fields for each sample acquired with constant parameters. (**C**) Representative immunofluorescence image of IGROV-1 tumor samples from mice treated with the anti-PD-1 antibody or untreated mice stained for IL-10 (green) and counterstained with 4′,6-diamidino-2-phenylindole (DAPI; blue) for nuclei. (**D**) Quantification of IL-10 positive area. Box and whiskers: min to max of data analyzing the positive area of five randomly selected fields for each sample acquired with constant parameters. *$p<0.05$ by Mann-Whitney U test. Original magnification 20x; inset magnification 40x. Bar: 100μm; Inset bar: 25μm. **anti-PD-1 Ab**: anti-PD-1 antibody.

Figure S3. Effect of the *in vitro* combination of CpG-ODNs and an anti-mouse PD-1 on CD80 and CD86 expression by RAW264.7 cells. RAW264.7 macrophages were seeded in 12-well plates and then stimulated with 1 μM CpG-ODNs and 10 μg/ml anti-mouse PD-1 antibody alone or in combination for 6 hours. Controls were left untreated. At the end of treatment, cells were collected and flow-cytometry analysis was performed to evaluate CD80 and CD86 expression. Representative scatter dot plot images showing the percentage of $CD80^+$ (**A**) and $CD86^+$ (**B**) RAW264.7 cells in the different experimental conditions. Mean Fluorescence Intensity for each experimental group was also indicated.

Figure S4. Effect of the combination of CpG-ODNs and an anti-mouse PD-1 antibody on macrophages *in vitro* and *in vivo*. (**A**) RAW264.7 macrophages were seeded in 12-well plates and then stimulated with 1 μM CpG-ODNs and 10 μg/ml anti-mouse PD-1 antibody

alone or in combination for 6 hours. Controls were left untreated. At the end of treatment, cells were collected and flow-cytometry analysis was performed to evaluate CD80 and CD86 expression. Representative scatter dot plot images showing the percentage of CD206^{+} RAW264.7 cells in the different experimental conditions. Mean Fluorescence Intensity for each experimental group was also indicated. (**B**-**E**) Mice xenografted with IGROV-1 human ovarian cancer cells were treated i.p. with CpG-ODNs (20 µg/mouse, 5 days/week for 2 weeks, starting seven days after tumor cell injection) alone or in combination with an anti-PD-1 antibody (clone RMP1-24) (delivered i.p., 200µg/mouse, 2 times/week for 2 weeks, starting seven days after tumor cell injection). (**A**) Representative immunofluorescence image of IGROV-1 tumor samples from mice treated with CpG-ODN or CpG-ODN in combination with anti-PD-1 antibody stained for CD206 (green) and counterstained with 4′,6-diamidino-2-phenylindole (DAPI; blue) for nuclei. (**B**) Quantification of CD206 positive area. Box and whiskers: min to max of data analyzing the positive area of five randomly selected fields for each sample acquired with constant parameters. (**C**) Representative immunofluorescence image of IGROV-1 tumor samples from mice treated with CpG-ODN or CpG-ODN in combination with anti-PD-1 antibody stained for IL-10 (green) and counterstained with 4′,6-diamidino-2-phenylindole (DAPI; blue) for nuclei. (**D**) Quantification of IL-10 positive area. Box and whiskers: min to max of data analyzing the positive area of five randomly selected fields for each sample acquired with constant parameters. *$p<0.05$ by Mann-Whitney U test. Original magnification 20x; inset magnification 40x. Bar: 100µm; Inset bar: 25µm. **CpG-ODNs**: CpG oligodeoxynucleotides; **anti-PD-1 Ab**: an-ti-PD-1 antibody.

Figure S5. PD-1 expression on RAW264.7 cells and its modulation by TLR9 stimulation. Representative histogram plot of PD-1 expression on the RAW264.7 macrophages treated in vitro with 1 µM CpG-ODNs or left untreated. MFI: mean fluorescence intensity. CpG-ODNs: CpG oligodeoxynucleotides.

Figure S6. Anti-PD-1 antibody F(ab)$_2$ retains the ability to bind PD-1. EL4 mouse lymphoma cells, constitutively expressing high levels of PD-1, were stained with anti-PD-1 antibody (clone RMP1-14, green line) or with anti-PD-1 antibody F(ab)$_2$ (red line). FITC-conjugated secondary antibody stained cells served as controls (black line). Histogram overlay demonstrates that anti-PD-1 antibody F(ab)$_2$ is still able to bind PD-1 on EL-4 cells.

Figure S7. Modulation of FcγR2b and FcγR3 expression on RAW264.7 macrophages in vitro by the combination of CpG-ODNs and an anti-mouse PD-1 antibody. RAW264.7 macrophages were seeded in 12-well plates and then stimulated with 1 µM CpG-ODN and 10 µg/ml anti-mouse PD-1 antibody alone or in combination for 6 hours. Controls were left untreated. At the end of treatment, mRNA was extracted, and real-time PCR was performed. The data were normalized to the expression of β2m as a housekeeping reference gene and analyzed by the comparative $2^{-\Delta Ct}$ method. The data are presented as the mean ± SEM. ***$p<0.001$ by one-way ANOVA followed by Tukey's multiple comparison test with a single pooled variance or by the Kruskal-Wallis test followed by Dunn's multiple comparison test for nonparametric data. CpG-ODNs: CpG oligodeoxynucleotides; anti-PD-

1 Ab: anti-PD-1-Ab.

Figure S8. Evaluation of the ability of in vitro stimulated-BMDMs to modulate NK cell cytotoxic activity. BMDMs were stimulated with CpG-ODNs, anti-mouse PD-1 antibody, anti-mouse PD-1 antibody Fc Silent™ alone or in combination for 6 hours. At the end of treatment, spleen-isolated NK cells were added to each experimental condition and co-cultured with BMDMs for 36 hours. NK cell cytotoxicity was evaluated by 51Cr release assay against YAC-1 target cells. Graph shows the percentage specific lysis of 51Cr-labeled YAC-1 cells by NK cells after 36 hours in co-culture with BMDMs pre-incubated for 6 hours as indicated in the x-axis. The data are presented as the mean ± SEM. ***$p<0.001$ by one-way ANOVA followed by Tukey's multiple comparison test with a single pooled variance. **CpG-ODNs**: CpG oligodeoxynucleotides; **anti-PD-1 Ab**: anti-PD-1-Ab.

Figure S9. Effect of CpG-ODNs in combination with different concentrations of anti-mouse PD-1 antibody on RAW264.7 macrophages in vitro. RAW264.7 macrophages were seeded in 12-well plates and then stimulated with 1 µM CpG-ODN alone or in combination with decreasing concentrations of anti-mouse PD-1 antibody (ranging from 10 to 0.312 µg/ml) for 6 hours. Controls were left untreated. At the end of treatment, mRNA was extracted, and real-time PCR was performed. The data were normalized to the expression of β2m as a housekeeping reference gene and analyzed by the comparative $2^{-\Delta Ct}$ method. The data are presented as the mean ± SEM. Black line represents the mRNA induction of the indicated gene in CpG-ODN-treated RAW264.7 cells. **CpG-ODNs**: CpG oligodeoxynucleotides; **anti-PD-1 Ab**: anti-PD-1-Ab.

Table S1. List of TaqMan® gene expression assays utilized for Real Time PCR analysis.

Table 2. Effect of CpG-ODN and anti-PD-1 antibody combination on ascites appearance in IGROV-1 tumor-bearing mice. Athymic nude mice were xenografted with IGROV-1 human ovarian cancer cells and treated with CpG-ODNs, an anti-PD-1 antibody or their combination on different treatment schedules. The number in each bracket in the first column indicates the number of days between tumor cell injection and the administration of the first treatment.
