# Supplementary material for: Macrophages Impair TLR9 Agonist Antitumor Activity through Interacting with the Anti-PD-1 Antibody Fc Domain
Source: Cancers (Basel). 2021 Aug 13;13(16):4081. doi: 10.3390/cancers13164081 (PMC8391891; doi:10.3390/cancers13164081)
Supplement: Supplementary file 1 [file cancers-13-04081-s001.zip › Table S1.pdf]

Gene Symbol	Assay ID
β2m	Mm00437762_m1
Ccl1	Mm00441236_m1
Ccl2	Mm00441242_m1
Cd80	Mm00711660_m1
Cd86	Mm00444540_m1
Fcgr1	Mm00438874_m1
Fcgr2b	Mm00438875_m1
Fcgr3	Mm01290525_m1
Fcgr4	Mm07300046_g1
Il1b	Mm00434228_m1
Il6	Mm00446190_m1
Il12b	Mm00434174_m1
Il-10	Mm01288386_m1
Irf4	Mm00516431_m1
Irf5	Mm00496477_m1
Mertk	Mm00434920_m1
Nos2	Mm00440502_m1
Pdcd1	Mm00435532_m1
Socs1	Mm00782550_s1
Socs2	Mm05820064_g1
Stat1	Mm01257286_m1
Stat6	Mm01160477_m1
Tnf-α	Mm00443258_m1

Table S1. List of TaqMan® gene expression assays utilized for Real Time PCR analysis
