# Supplementary material for: Macrophages Impair TLR9 Agonist Antitumor Activity through Interacting with the Anti-PD-1 Antibody Fc Domain
Source: Cancers (Basel). 2021 Aug 13;13(16):4081. doi: 10.3390/cancers13164081 (PMC8391891; doi:10.3390/cancers13164081)
Supplement: Supplementary file 1 [file cancers-13-04081-s001.zip › Table S2.pdf]

A (data related to Figure 2A)		
Experimental Group	N° of mice with ascites†	Time of ascites appearance (Median of days)
Control	7/7 (100%)	9
Anti-PD-1 Ab	7/7 (100%)	9
CpG-ODN (+7)	1/7 (14.29%)	Undefined
CpG-ODN (+7) + Anti-PD-1 Ab (+7)	6/7 (85.71%)	20
CpG-ODN (+7) + Anti-PD-1 Ab (+13)	4/7 (57.14%)	27
**B (data related to Figure 2B)**		
Control	7/7 (100%)	9
Anti-PD-1 Ab	7/7 (100%)	10
CpG-ODN (+7)	1/4 (25%)	Undefined
CpG-ODN (+7) + Anti-PD-1 Ab (+7)	4/5 (80%)	21

†total number of mice that develop ascites over the experiment

Table S2. Effect of CpG-ODN and anti-PD-1 antibody combination on ascites appearance in IGROV-1 tumor-bearing mice. Athymic nude mice were xenografted with IGROV-1 human ovarian cancer cells and treated with CpG-ODNs, an anti-PD-1 antibody or their combination on different treatment schedules. The number in each bracket in the first column indicates the number of days between tumor cell injection and the administration of the first treatment.
