# Supplementary figures and images for: Macrophages Impair TLR9 Agonist Antitumor Activity through Interacting with the Anti-PD-1 Antibody Fc Domain
Source: Cancers (Basel). 2021 Aug 13;13(16):4081. doi: 10.3390/cancers13164081 (PMC8391891; doi:10.3390/cancers13164081)

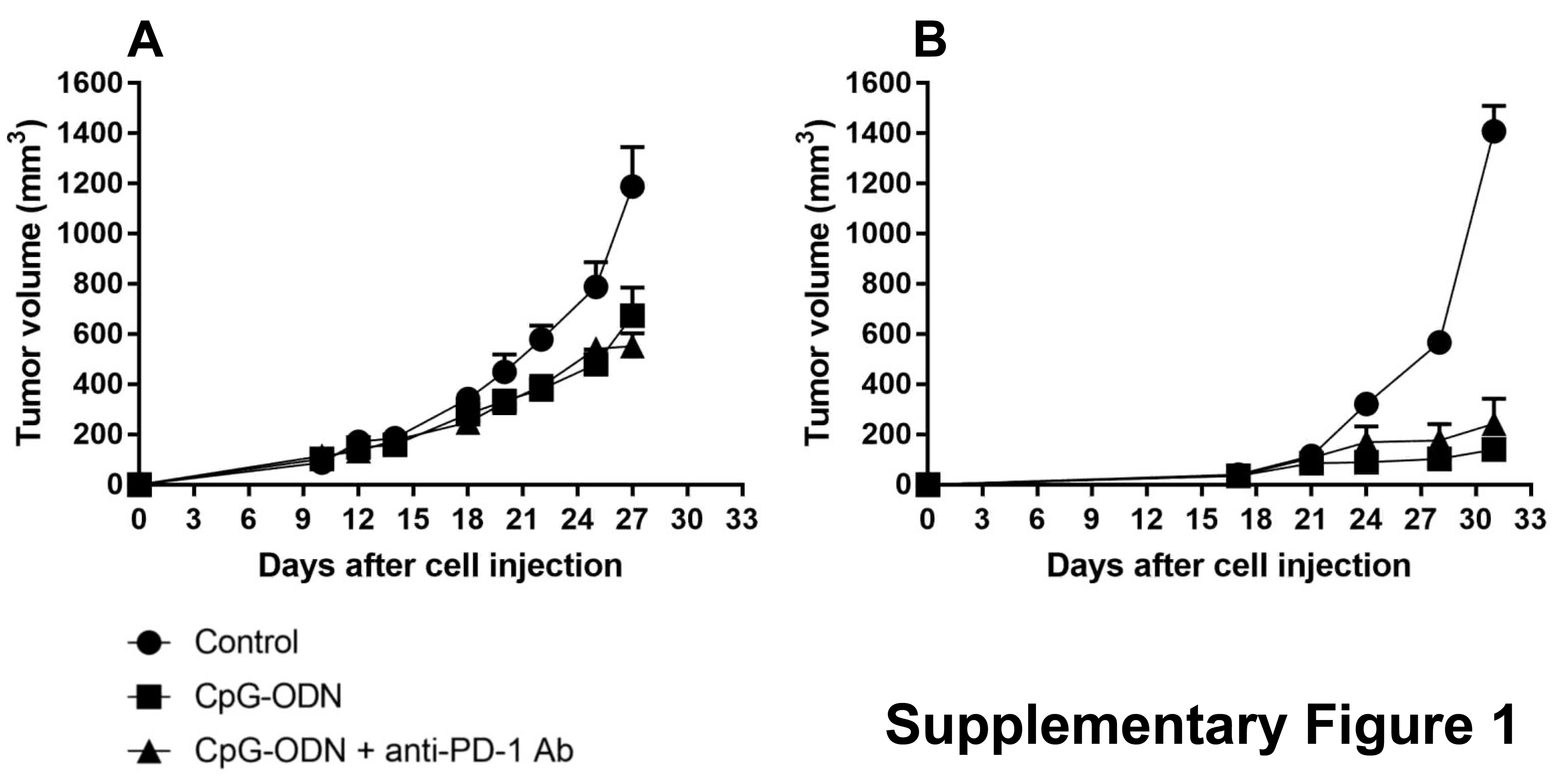

Supplement: Supplementary file 1 [file cancers-13-04081-s001.zip › Figure S1.tif]

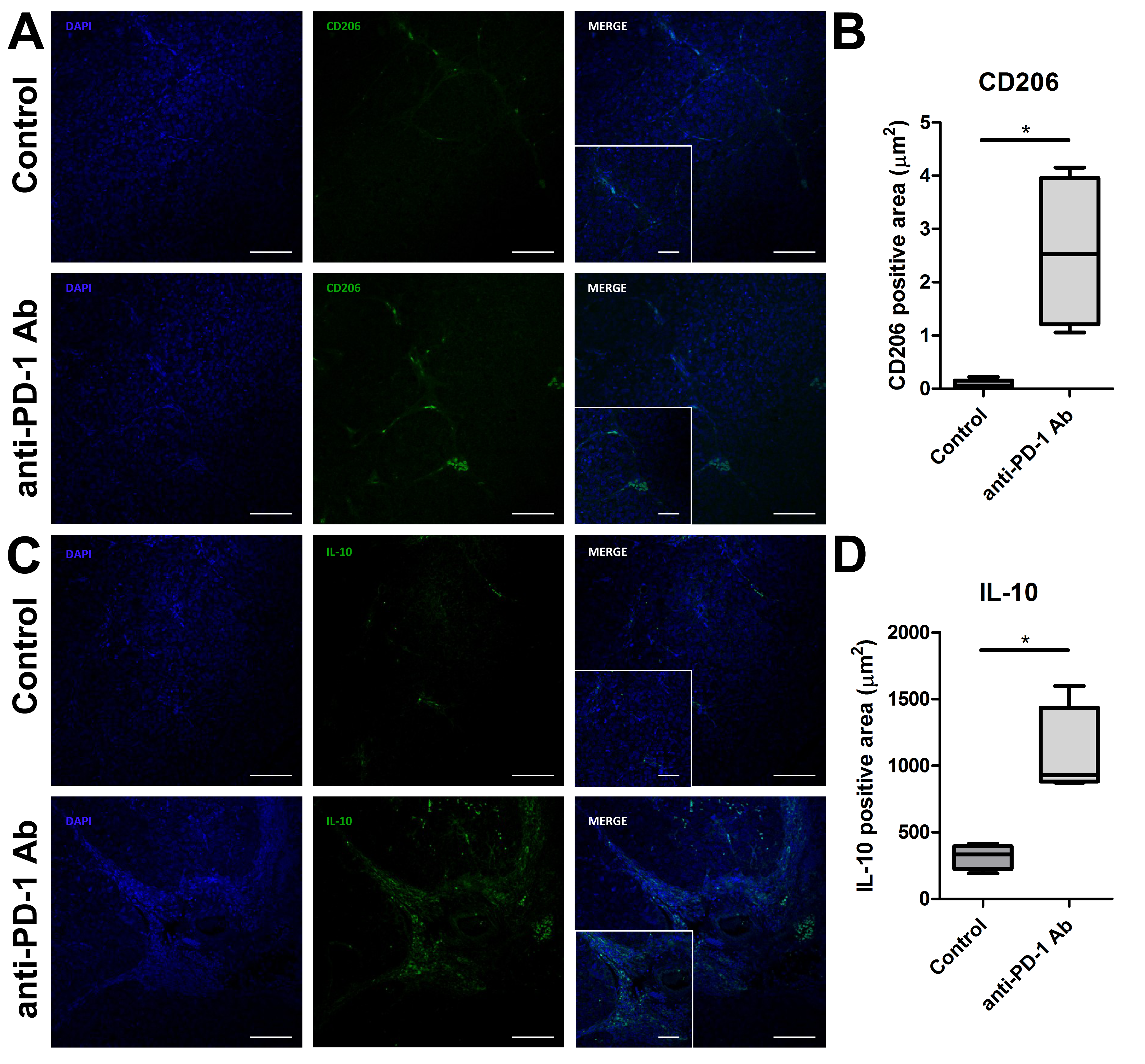

Supplement: Supplementary file 1 [file cancers-13-04081-s001.zip › Figure S2.tif]

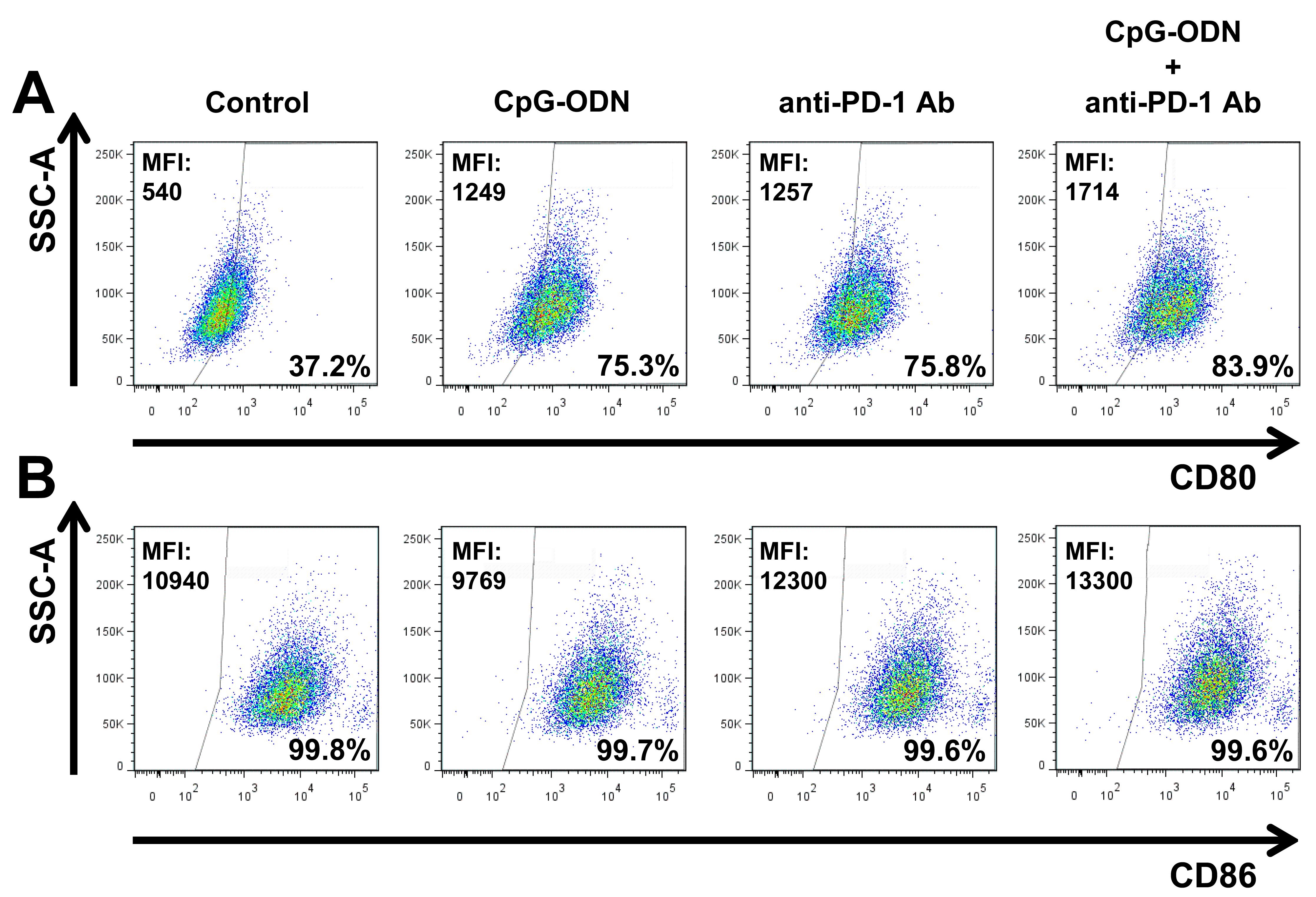

Supplement: Supplementary file 1 [file cancers-13-04081-s001.zip › Figure S3.tif]

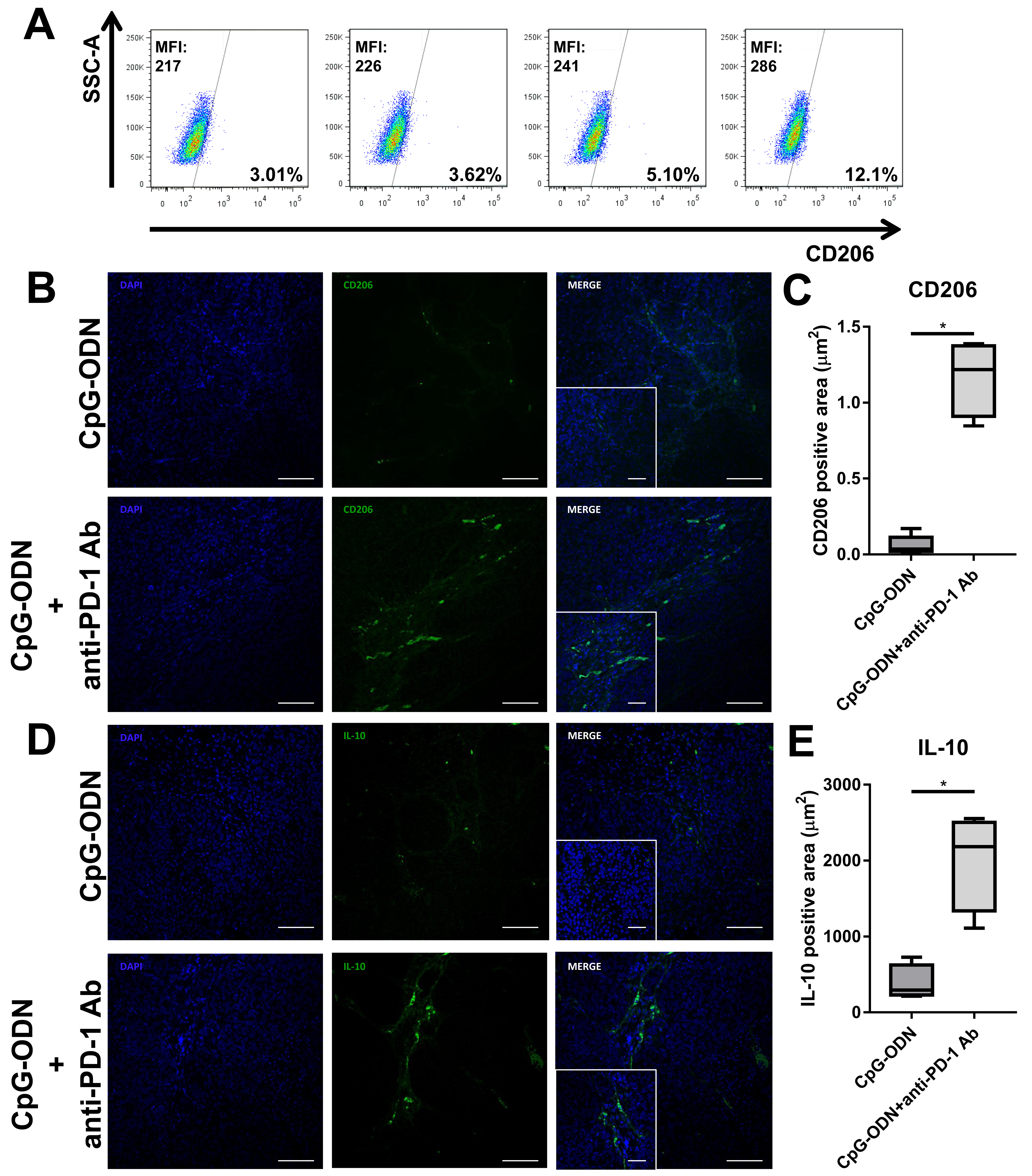

Supplement: Supplementary file 1 [file cancers-13-04081-s001.zip › Figure S4.tif]

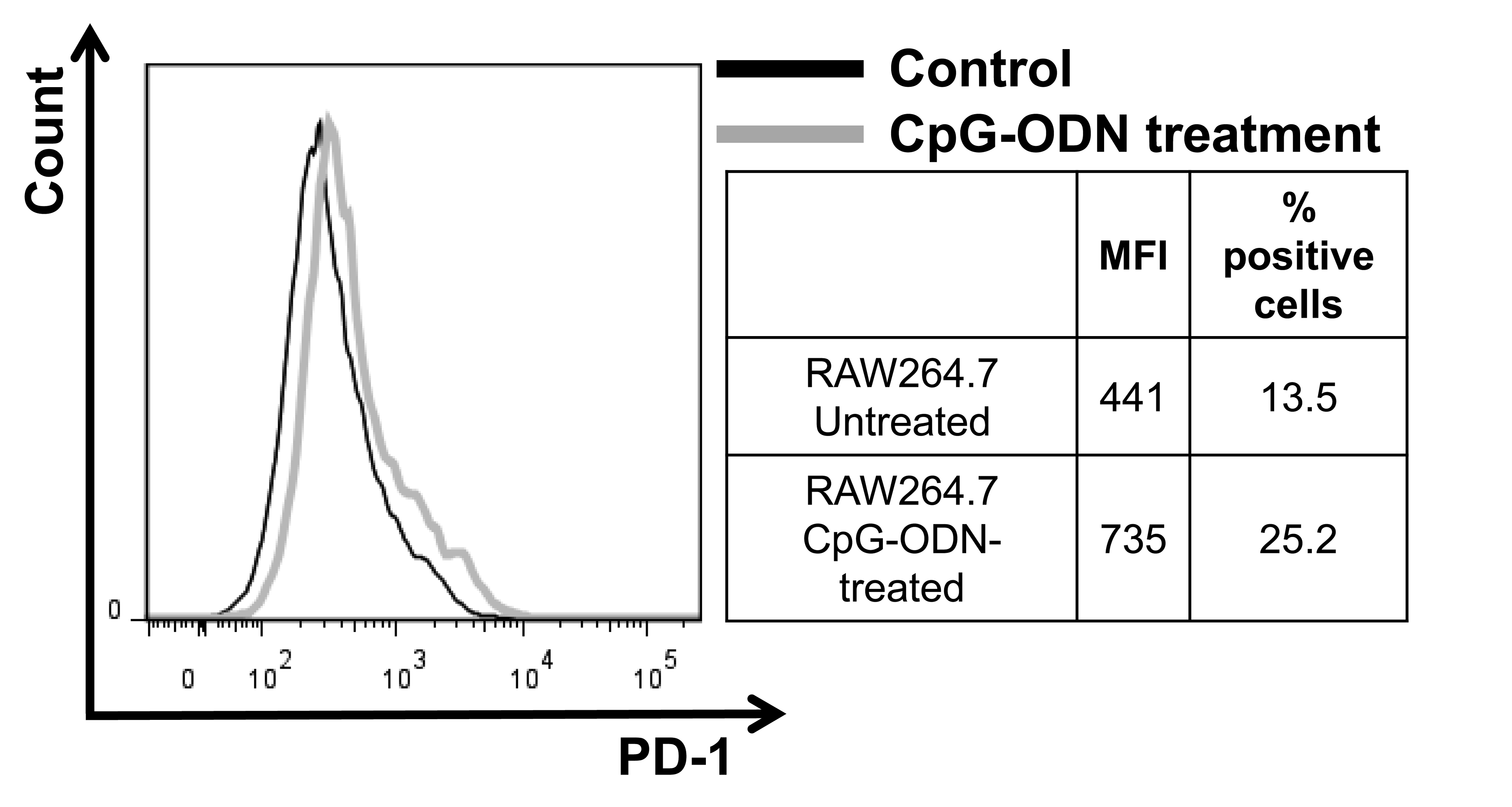

Supplement: Supplementary file 1 [file cancers-13-04081-s001.zip › Figure S5.tif]

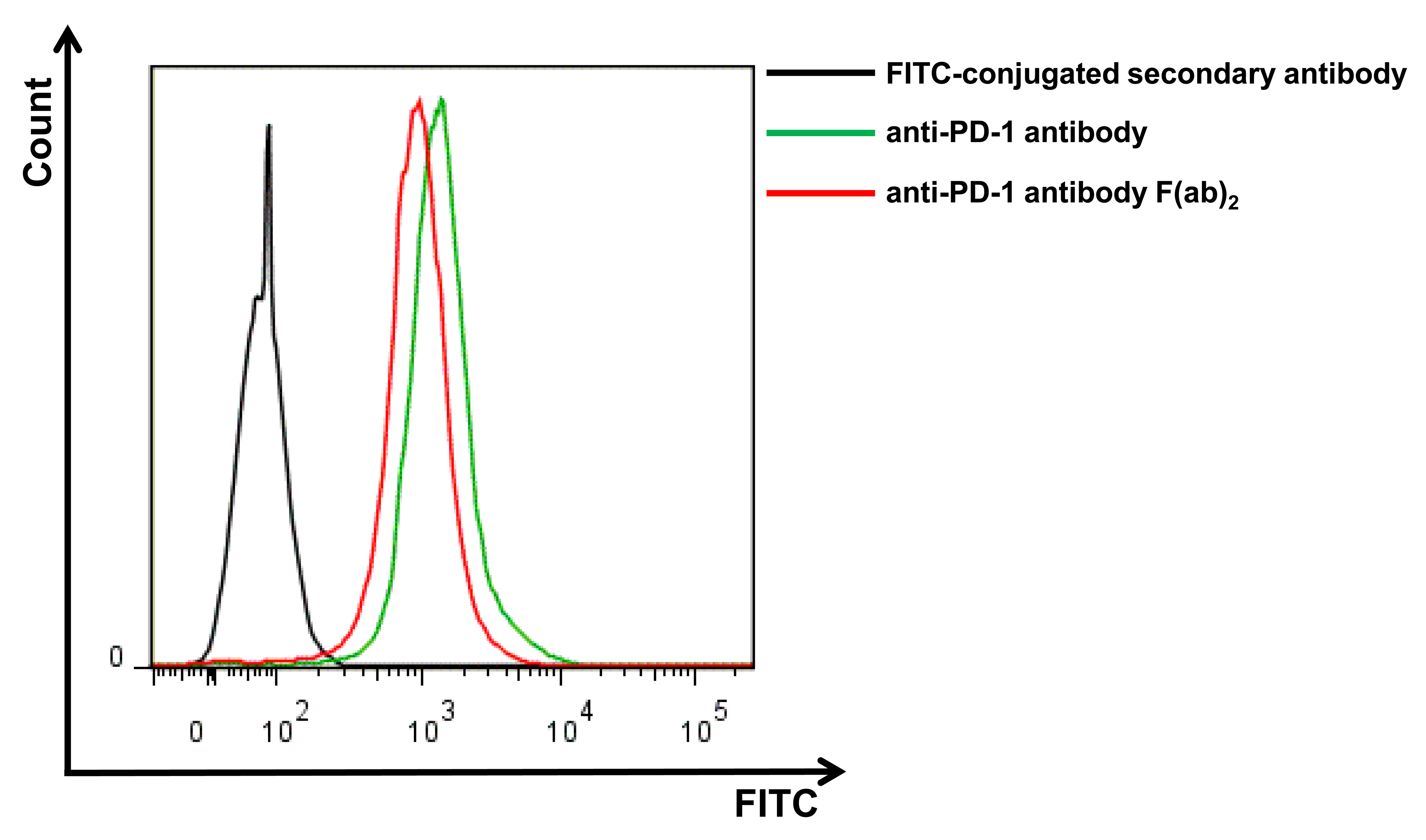

Supplement: Supplementary file 1 [file cancers-13-04081-s001.zip › Figure S6.tif]

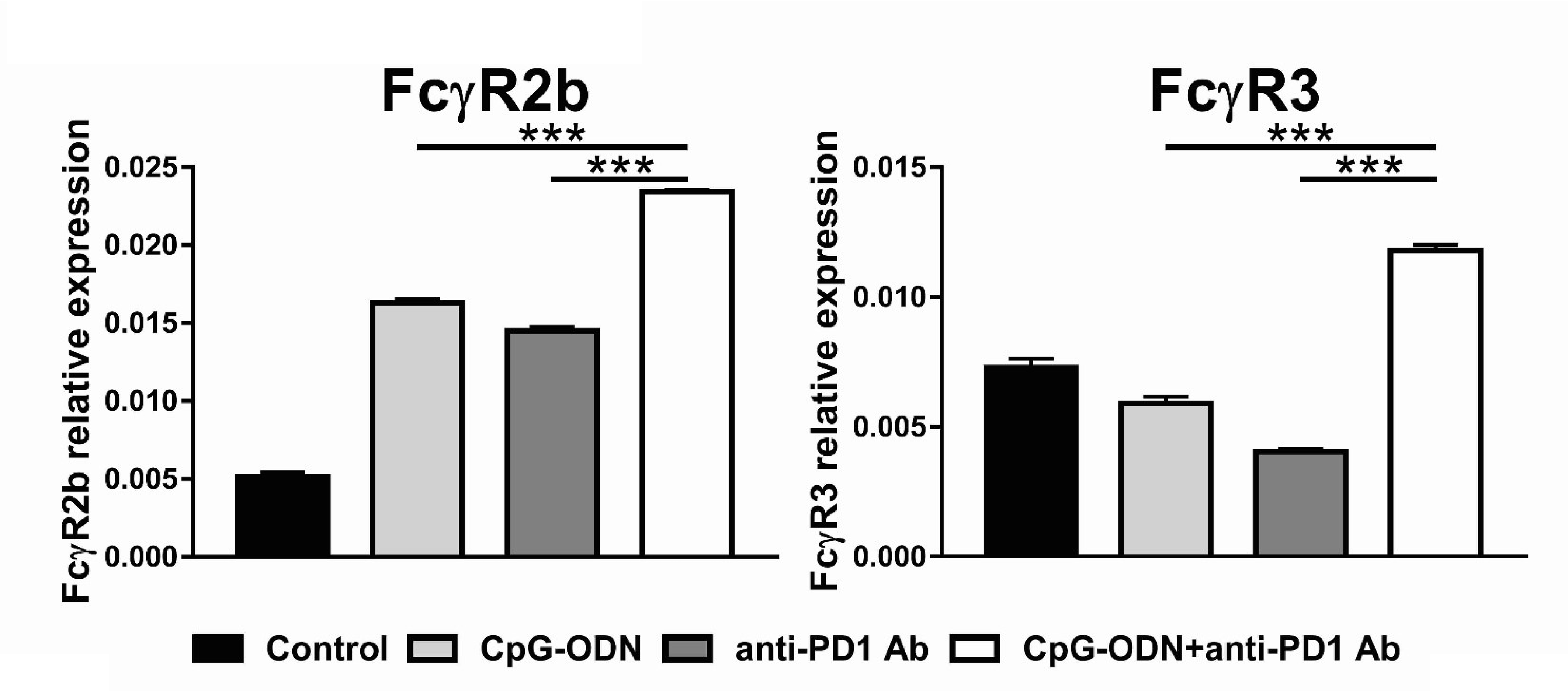

Supplement: Supplementary file 1 [file cancers-13-04081-s001.zip › Figure S7.tif]

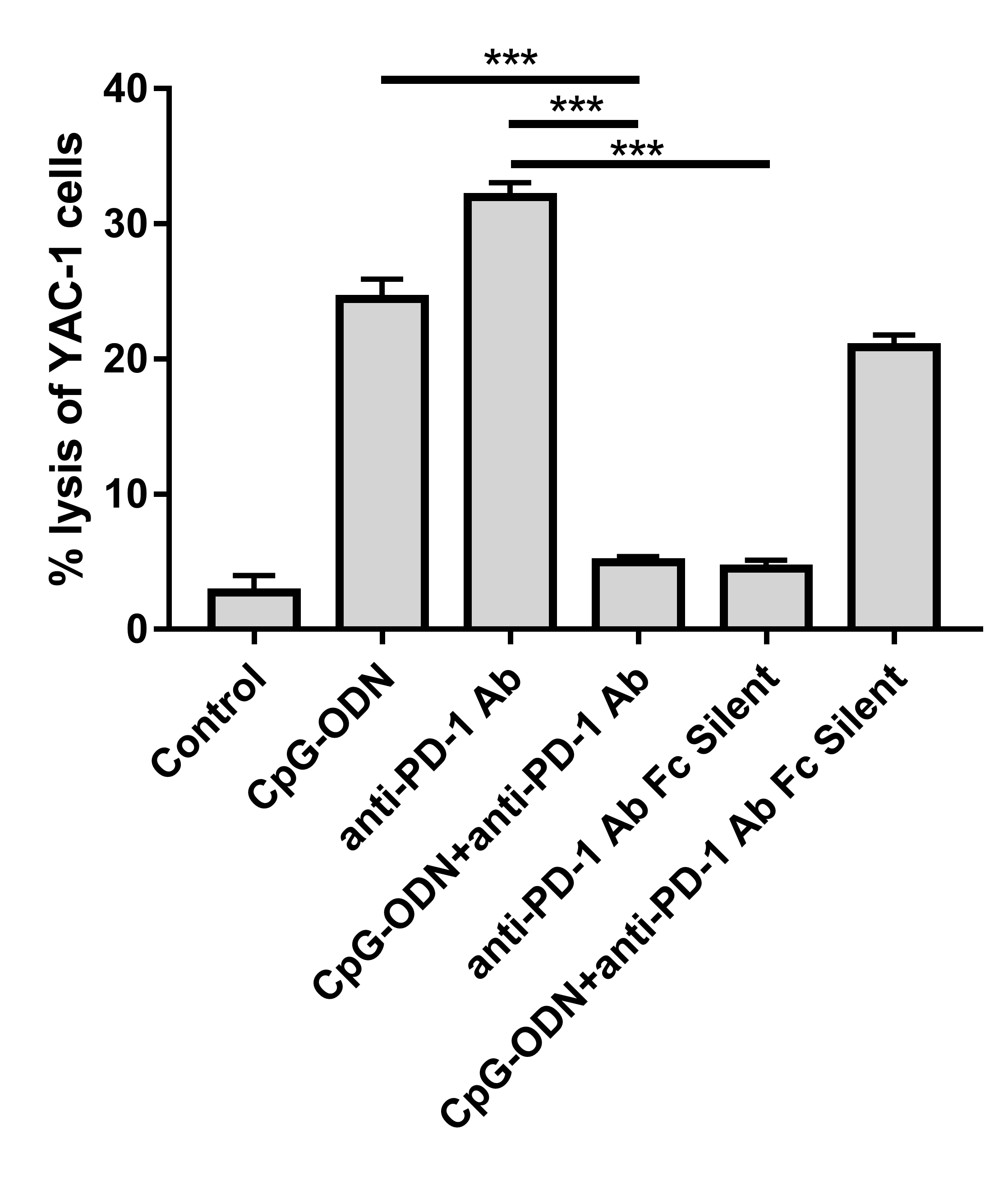

Supplement: Supplementary file 1 [file cancers-13-04081-s001.zip › Figure S8.tif]
